# Supplementary material for: Identification of Freezing-Responsive microRNAs and Their Targets in Chinese Jujube by Small RNA and Degradome Sequencing
Source: Int J Mol Sci. 2026 Sep 4;27(17):7912. doi: 10.3390/ijms27177912 (PMC13566890; doi:10.3390/ijms27177912)
Supplement: Supplementary file 1 [file ijms-27-07912-s001.zip › Supplementary Material S2/novel_miR_114.pdf]

Provisional ID	:	novel_miR_114
Score total	:	202245.3
Score for star read(s)	:	3.9
Score for read counts	:	202239.1
Score for mfe	:	0.7
Score for randfold	:	1.6
Score for cons. seed	:	
Total read count	:	396695
Mature read count	:	396569
Loop read count	:	3
Star read count	:	123

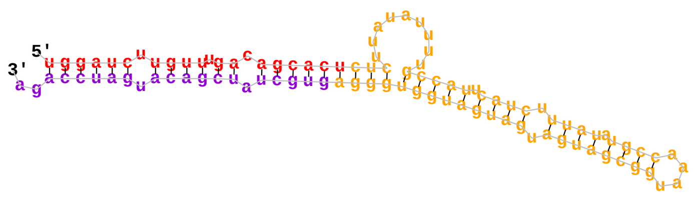

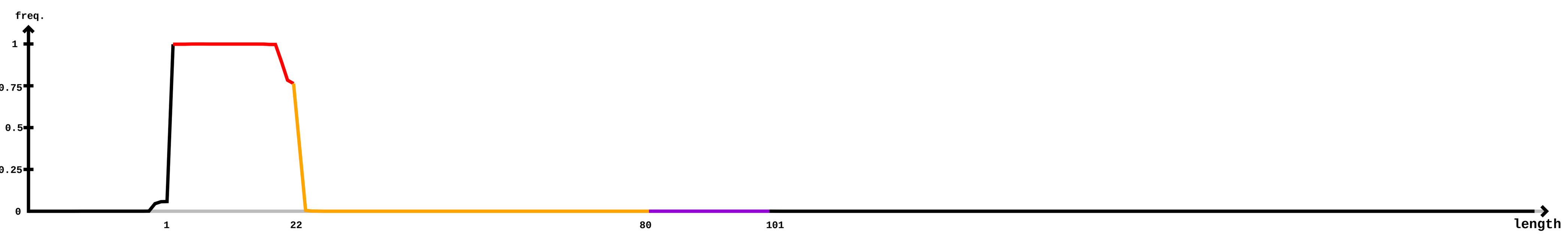

Mature **Star**

```
5'- uccaucuuguucguuuacccucuggaucuuguuugacagcacucucuuauauuuugccauucaucuuuauaugccaaauggcgaugaugaugaugguggga gugcuaucgacaugauccagaaggugcagucgaauaagauacagagggaccacccacaguaccuacucucguuacaaucguauucuucuccucacuuauuauuauuauuauuauuauuauuccgaucauuuucuuuuaaucuauugaag -3' obs
    uccaucuuguucguuuacccucuggaucuuguuugacagcacucucuuauauuuugccauucaucuuuauaugccaaauggcgaugaugaugaugguggagugcuaucgacaugauccagaaggugcagucgaauaagauacagagggaccacccacaguaccuacucucguuacaaucguauucuucuccucacuuauuauuauuauuauuauuauuauuccgaucauuuucuuuuaaucuauugaag     exp
    ...((((((((((((((.((((((((.((((.((.(((((((((........(((((.((((.((((.((((....)))))))).))))))))))))))))).)))))).)))))))).)))).)).))))))))))((((((((((........)).))..)))).))...................   reads  mm  sample
```

Read	reads	mm	sample
.ccaucuuguucguuuacc........	1	0	LT-1
.ccaucuuguucguuuacccucugg........	5	0	LCK-3
.ccaucuuguucguuuacccucuggauc........	1	0	LT-3
...aucuuguucguuuacccucugga........	2	0	JCK-1
.....cuuguucguuuacccucuggauc........	4	0	LT-2
......uuguucguuuaccUucuggauc........	2	1	JCK-1
......uuguucguuuaccUucuggauc........	6	1	JCK-2
......uuguucguuuaccUucuggauc........	2	1	LT-2
......uuguucguuuaccUucuggauc........	3	1	JCK-3
.......uguucguuuaccUucuggauc........	12	1	LT-1
.......uguucguuuaccUucuggauc........	30	1	JCK-3
.......uguucguuuacccucuggauc........	2	0	JCK-3
.......uguucguuuacccucuggauc........	1	0	JCK-2
.......uguucguuuaccUucuggauc........	31	1	JCK-2
.......uguucguuuacccucuggauc........	1	0	JCK-1
.......uguucguuuaccUucuggauc........	3	1	LT-2
.......uguucguuuaccUucuggauc........	10	1	JCK-1
.......uguucguuuaccUucuggauc........	12	1	LT-3
..........ucguuuacccucuggaucuug........	1	0	LT-2
............guuuacccucuggaucuuguuugaca........	2	0	LCK-3
.............uuuacccucuggaucuuguA........	1	1	JCK-1
.............uuuacccucuggaucuuguuu........	2	0	JCK-3
.............uuuacccucuggaucuuguuuga........	1	0	JCK-1
...............uacccucuggaucuuguuugacagc........	2	0	JCK-2
................Ccccucuggaucuuguuuga........	1	1	LT-1
................acccucuggaucuuguuugacagc........	2	0	JCK-2
................acccucuggaucuuguuugacagca........	2	0	JCK-2
................acccucuggaucuuguuugacagca........	2	0	JCK-3
.................cccucuggaucuuguuugacagc........	1	0	LT-3
..................ccucuggaucuuguuugac........	3	0	JCK-2
..................ccucuggaucuuguuugaca........	2	0	JCK-3
..................ccucuggaucuuguuugaca........	2	0	JCK-1
..................ccucuggaucuuguuugacag........	1	0	JCK-3

Mature **Star**

uccaucuuguucguuuacccucuggaucuuguuugacagcacucucuuauauuuugccauucaucuuuauaugccaaauggcgaugaugaugauggugggagugcuaucgacaugauccagaaggugcagucgaauaagauacagagggaccacccacaguaccuacucucguuacaaucguauucuucuccucacuuauuauuauuauuauuauuauuccgaucauuuucuuuuaaucuauugaag

..................ccucuggaucuuguuugacag	4	0	LCK-3
..................ccucuggaucuuguuugacag	1	0	JCK-1
..................ccucuggaucuuguuugacag	1	0	JCK-2
..................ccucuggaucuuguuugacagc	9	0	LT-3
..................ccucuggaucuuguuugacagc	3	0	LT-2
..................ccucuggaucuuguuugacagc	35	0	JCK-3
..................ccucuggaucuuguuugacagc	21	0	JCK-1
..................ccucuggaucuuguuugacagc	6	0	LCK-3
..................ccucuggaucuuguuugacagc	32	0	JCK-2
..................ccucuggaucuuguuugacagc	6	0	LT-1
..................ccucuggaucuuguuugacagcU	2	1	JCK-1
..................ccucuggaucuuguuugacagcU	4	1	JCK-2
..................ccucuggaucuuguuugacagcU	1	1	JCK-3
...................cucuggaucuuguuugac	8	0	LT-1
...................cucuggCucuuguuugac	1	1	JCK-2
...................cucuggaucuuguuugac	6	0	LCK-3
...................cucuggaucuuguuugaU	2	1	JCK-3
...................cucuggaucuuguuugac	13	0	JCK-2
...................cucuggaucuuguuugac	13	0	LT-2
...................cucuggaucuuguuugac	13	0	LT-3
...................cucuggaucuuguuugac	10	0	JCK-3
...................cucuggaucuuguuugac	4	0	JCK-1
...................cucuggaucuuguuugaca	78	0	LCK-3
...................cucuggaucuuguuugaca	27	0	LT-1
...................cucuggaucuuguuugaca	29	0	LCK-2
...................cucuggaucuuguuugaca	18	0	JT-2
...................cucuggaucuuguuugacC	1	1	LT-1
...................cucuggaucuuguuugaca	20	0	JCK-1
...................cucuggaucuuguuugacU	2	1	LT-1
...................cucuggaucuuguuugaUa	1	1	JCK-2
...................cucuggaucuuguuugaca	18	0	JCK-3
...................cucuggaucuuguuugaca	62	0	LT-2
...................cucuggaucuuguuugacU	6	1	LT-2
...................cucuggaucuuguuugacU	6	1	JCK-3
...................cucuggaucuuguuugaca	21	0	JT-3
...................cucuggaucuuguuugacU	1	1	LT-3
...................cucuggaucuuguuugaca	42	0	LCK-1
...................cucuggaucuuguuugacU	14	1	LCK-3
...................cucuggCucuuguuugaca	1	1	LCK-1
...................cucuggaucuuguuugaca	28	0	JT-1
...................cucuggCucuuguuugaca	1	1	JT-1
...................cucuggaucuuguuugaca	65	0	LT-3
...................cucuggaucuuguuugacU	7	1	JCK-2
...................cucuggaucuuguuugaUa	1	1	JCK-3
...................cuNuggaucuuguuugaca	1	1	LT-2
...................cucuggaucuuguuugaca	41	0	JCK-2
...................cucuggaucuuguuugacag	6	0	JCK-3
...................cucuggaucuuguuugacag	12	0	LT-2
...................cucuggaucuuguuugacag	5	0	JCK-1
...................cucuggaucuuguuugacag	14	0	JT-2
...................cucuggaucuuAuuugacag	1	1	LT-2
...................cucuggaucuuguuugacag	7	0	LT-1
...................cucuggaucuuguuugacag	15	0	JT-3
...................cucuggaucuuguuugacaU	2	1	LT-1
...................cucuggaucuuguuugacag	23	0	LCK-3
...................cucuggaucuuguuugacaU	3	1	LT-3
...................cucuggaucuuguuugacag	7	0	LT-3
...................cucuggaucuuguuugacag	4	0	JCK-2
...................cucuggCucuuguuugacagc	4	1	JCK-3
...................cucuggaucuuguuugacagc	292	0	LCK-2
...................cucuggaucuuguuugacagc	1675	0	LT-2
...................cucuggaucuuguuugacagU	2	1	LT-3
...................cucuggaucuuguuugacagc	3319	0	LT-1
...................cucCggaucuuguuugacagc	2	1	JCK-3
...................cucuggCucuuguuugacagc	11	1	JCK-2
...................cucCggaucuuguuugacagc	3	1	LT-3
...................cucuggaucuuguCugacagc	1	1	JCK-2
...................cucuggauUuuguuugacagc	1	1	LT-1
...................cucugNaucuuguuugacagc	1	1	LCK-1
...................cucuggaCcuuguuugacagc	2	1	LT-1

Mature Star

uccaucuuguucguuuacccucuggaucuuguuugacagcacucucuuauauuuugccauucaucuuuauaugccaaauggcgaugaugaugauggugggagugcuaucgacaugauccagaaggugcagucgaauaagauacagagggaccacccacaguaccuacucucguuacaaucguauucuucuccucacuuauuauuauuauuauuauuauuccgaucauuucuuuuaaucuauugaag

cCcuggaucuuguuugacagc	1	1	JCK-2
cucuggaucuCguuugacagc	1	1	LT-1
cucuggCucuuguuugacagc	1	1	JCK-1
cucuggaucuuguuugCcagc	4	1	JCK-1
cucuggaucuuguuugacagc	1379	0	LCK-3
cucuggaucuuNuuugacagc	3	1	JCK-1
cucuggaucuuguuugacagU	1	1	JCK-3
cuUuggaucuuguuugacagc	2	1	JCK-1
cNcuggaucuuguuugacagc	2	1	JCK-1
cucCggaucuuguuugacagc	1	1	LT-1
cucuggaucuuguuuAacagc	1	1	LT-1
cucuggaucuuguuuAacagc	1	1	LCK-3
cucuggauNuuguuugacagc	2	1	JCK-3
cucuggaucuugCuugacagc	1	1	JCK-2
cucuggaucuuguuugacagc	2907	0	JCK-2
cucuggaucuuguuugacCgc	2	1	JCK-3
cucuggaucuuguuugacaNc	4	1	JCK-1
cucuggaucuuAuuugacagc	1	1	LT-1
cNcuggaucuuguuugacagc	1	1	JCK-3
cucuggaucuuguuugacagc	3001	0	LT-3
cucuggauAuuguuugacagc	1	1	LT-1
cucuUgaucuuguuugacagc	1	1	LT-3
cucuggaucuuguuugacGgc	1	1	LT-3
cucuggaucuuguuugacCgc	2	1	JCK-1
cucGggaucuuguuugacagc	1	1	JCK-1
cucuggCucuuguuugacagc	1	1	LCK-2
cucuggaucuuguuugacagc	90	0	JT-3
cuUuggaucuuguuugacagc	3	1	JCK-3
cucuAgaucuuguuugacagc	1	1	JCK-2
cucuggaucCuguuugacagc	1	1	JCK-2
cucuggaucuuguuugaGagc	1	1	JCK-1
cucuggaucCuguuugacagc	1	1	LT-1
cucuggaucuuguuugacCgc	2	1	LT-1
cucuggaucuuAuuugacagc	1	1	LT-3
cucugAaucuuguuugacagc	1	1	LT-3
cucuAgaucuuguuugacagc	1	1	JCK-1
cucuggaucuuguuugacagc	1680	0	JCK-1
cucuggCucuuguuugacagc	11	1	LT-1
cNcuggaucuuguuugacagc	1	1	LCK-1
cucuggaucuuguuugGcagc	1	1	JCK-3
cuAuggaucuuguuugacagc	2	1	JCK-3
cuNuggaucuuguuugacagc	2	1	LT-2
cucCggaucuuguuugacagc	2	1	JCK-2
cucuggaucuuguuugacCgc	2	1	JCK-2
cucCggaucuuguuugacagc	1	1	LT-2
cucuggaucuuguuugacagc	66	0	JT-1
cucugUaucuuguuugacagc	1	1	LT-1
cNcuggaucuuguuugacagc	1	1	LT-3
cGcuggaucuuguuugacagc	1	1	LT-2
cucuggaucuuguuugaUagc	2	1	JCK-3
cCcuggaucuuguuugacagc	1	1	LT-1
cNcuggaucuuguuugacagc	4	1	JCK-2
cucuggaucuuAuuugacagc	2	1	JCK-2
cucuggaucuuguuugCcagc	9	1	LT-1
cucuggaucuuguuugacagA	3	1	LT-2
cucuggaucuuguuugacagc	2088	0	JCK-3
cucuggaucuuUuuugacagc	1	1	LT-1
cucuggaucuuguuugCcagc	7	1	LT-2
cucuUgaucuuguuugacagc	1	1	LT-2
cCcuggaucuuguuugacagc	2	1	LT-2
cucuggaucuuguuugCcagc	5	1	LT-3
cucuggaucuuUuuugacagc	1	1	JCK-3
cCcuggaucuuguuugacagc	1	1	LCK-2
cucuggaucuuguuugCcagc	2	1	LCK-3
cucuggCucuuguuugacagc	11	1	LT-3
cucuggaucuuguuugaUagc	2	1	JCK-2
cucuggaucuuguuugacCgc	2	1	LCK-3
cucuggaucuuguuugacCgc	4	1	LT-3
cucuAgaucuuguuugacagc	1	1	LCK-3
cucuggaucuCguuugacagc	1	1	LCK-3

```
                    Mature                                                                       Star
uccaucuuguucguuuacccucuggaucuuguuugacagcacucucuuauauuuugccauucaucuuuauaugccaaauggcgaugaugaugauggugggagugcuaucgacaugauccagaaggugcagucgaauaagauacagagggaccacccacaguaccuacucucguuacaaucguauucuucuccucacuuauuauuauuauuauuauuauuccgaucauuuucuuuuaaucuauugaag
...................cucugAaucuuguuugacagc        3     1    LT-2
...................cucuggaucuuguuugacCgc        2     1    LT-2
...................cCcuggaucuuguuugacagc        1     1    LCK-3
...................cucuggaucuuguCugacagc        1     1    JCK-3
...................cucugAaucuuguuugacagc        1     1    JCK-3
...................cucuAgaucuuguuugacagc        3     1    LT-3
...................cucuggaucuuguuuUacagc        1     1    JCK-2
...................cucuggCucuuguuugacagc        4     1    LCK-3
...................cucuggaucuuguuugCcagc        3     1    JCK-2
...................cucuggCucuuguuugacagc        20    1    LT-2
...................cucNggaucuuguuugacagc        2     1    JCK-1
...................cuGuggaucuuguuugacagc        1     1    LT-2
...................cucuggaucuCguuugacagc        2     1    LT-2
...................cucuggaucuuguuugacagc        275   0    LCK-1
...................cucuAgaucuuguuugacagc        4     1    LT-2
...................cucuggaucuuguuugCcagc        1     1    JCK-3
...................cucuggaucuuguuugaUagc        1     1    LT-3
...................cucuggaucuuguuugacagc        62    0    JT-2
...................cucuggCucuuguuugacagc        2     1    LCK-1
...................cucuggaucuuguuugacagcC       2     1    LT-1
...................cucuggaucuuguuugacagcU       14    1    JT-3
...................cucuggaucuuguuugacagcU       29    1    JCK-3
...................cucuggaucuuguuugacagcU       47    1    LT-2
...................cucuggaucuuguuugacagca       87    0    JCK-3
...................cucuggaucuuguuugacagcU       69    1    LT-3
...................cucuggaucuuguuugacagca       10    0    JT-3
...................cucuggaucuuguuugacagca       72    0    JCK-1
...................cucuggaucuuguuugacagca       19    0    LT-2
...................cucuggaucuuguuuNacagca       1     1    JCK-3
...................cucuggaucuuguuugacagcC       2     1    LT-2
...................cucuggaucuuguuugacagca       117   0    JCK-2
...................cucuggaucuuguuugacagca       22    0    LT-3
...................cucuggaucuuguuugacagca       1     0    JT-2
...................cucuggaucuuguuugacagcC       1     1    JCK-2
...................cucuggaucuuguuugacagcU       84    1    LCK-3
...................cucuggaucuuguuugacagca       27    0    LT-1
...................cucuggaucuuguuugacagcG       1     1    JT-2
...................cucuggaucuuguuugacagcU       13    1    JT-2
...................cucuggaucuuguuugacagcU       66    1    LT-1
...................cucuggaucuuguuugacagcU       25    1    JCK-1
...................cucuggaucuugCuugacagca       1     1    JCK-2
...................cucuggaucuuguuugacagcC       2     1    LT-3
...................cucuggaucuuguuugacagcU       55    1    JCK-2
...................cucuggaucuuguuugacagca       5     0    LCK-3
...................cucuggaucuuguuugacagcac      1     0    LT-3
...................cucuggaucuuguuugacagcac      1     0    LT-1
...................cucuggaucuuguuugacagcacu     1     0    LT-2
...................cucuggaucuuguuugacagcacN     1     1    LT-2
....................ucuggaucuuguuugaca          28    0    LT-1
....................ucuggaucuuguuugaca          41    0    LT-2
....................ucuggaucuuguuugaca          26    0    LT-3
....................ucuggaucuuguuugaca          15    0    JCK-3
....................ucuggaucuuguuugaca          25    0    JCK-1
....................ucuggaucuuguuugaca          35    0    JT-3
....................ucuggaucuuguuugacU          2     1    LT-3
....................ucuggaucuuguuugacU          1     1    JCK-1
....................ucuggaucuuguuugaca          3     0    LCK-2
....................ucuggaucuuguuugaca          79    0    LCK-3
....................ucuggaucuuguuugacU          1     1    JCK-2
....................ucuggaucuuguuugaca          9     0    JT-1
....................ucuggaucuuguuugaca          30    0    JT-2
....................ucuggaucuuguuugacU          9     1    LCK-3
....................ucuggaucuuguuugacU          6     1    LT-1
....................ucuggaucuuguuugaca          33    0    JCK-2
....................ucuggaucuuguuugaca          3     0    LCK-1
....................ucuggaucuuguuugacag         2     0    LT-1
....................ucuggaucuuguuugacagc        55    0    JCK-2
....................ucuggCucuuguuugacagc        1     1    LCK-1
....................ucuggaucuuguuugacagc        35    0    JCK-3
....................ucuggaucuuguuuNacagc        1     1    LCK-1
```

```
                    Mature                                                                                       Star
uccaucuuguucguuuacccucuggaucuuguuugacagcacucucuuauauuuugccauucaucuuuauaugccaaauggcgaugaugaugauggugggagugcuaucgacaugauccagaaggugcagucgaauaagauacagagggaccacccacaguaccuacucucguuacaaucguauucuucuccucacuuauuauuauuauuauuauuauuccgaucauuuucuuuuaaucuauugaag
....................ucuggaucuuguuugacagc....................................................................................................................................................................................................    63    0    LCK-1
....................ucuggaucuuguuugacagA....................................................................................................................................................................................................    1     1    JCK-3
....................ucuggaucuuguuugacagc....................................................................................................................................................................................................    116   0    LT-2
....................ucuggaucuuguuugacagA....................................................................................................................................................................................................    1     1    JCK-1
....................ucuggaucuuguuugacagc....................................................................................................................................................................................................    127   0    LT-3
....................uGuggaucuuguuugacagc....................................................................................................................................................................................................    1     1    LCK-1
....................ucuggaucuuguuugacagc....................................................................................................................................................................................................    29    0    JCK-1
....................ucuggaucuuguuugacagc....................................................................................................................................................................................................    107   0    LT-1
....................ucuggCucuuguuugacagc....................................................................................................................................................................................................    2     1    LT-2
....................ucuggaucuuguuugacaUc....................................................................................................................................................................................................    1     1    LT-1
....................ucuggaucuuguuuUacagc....................................................................................................................................................................................................    1     1    LCK-2
....................ucuggaucuuguuugacagc....................................................................................................................................................................................................    75    0    LCK-2
....................ucuggaucuuguuugacagA....................................................................................................................................................................................................    1     1    LT-3
....................ucuggaucuuguuugacagc....................................................................................................................................................................................................    41    0    LCK-3
....................ucuggaucuuguuugacagcU...................................................................................................................................................................................................    14    1    LT-2
....................ucuggaucuuguuugacagca...................................................................................................................................................................................................    628   0    JCK-1
....................ucuggaucuuguuugacaUca...................................................................................................................................................................................................    5     1    JCK-2
....................ucuggaucuuguuugacagcU...................................................................................................................................................................................................    3     1    JCK-3
....................ucuggaucuuguuugacagca...................................................................................................................................................................................................    17    0    LCK-2
....................ucuggaucuuguuugacaUca...................................................................................................................................................................................................    2     1    JCK-1
....................ucuggaucuuguuugCcagca...................................................................................................................................................................................................    1     1    LT-1
....................uUuggaucuuguuugacagca...................................................................................................................................................................................................    1     1    JCK-1
....................ucuggaucuuguuugacagcU...................................................................................................................................................................................................    6     1    JCK-1
....................uUuggaucuuguuugacagca...................................................................................................................................................................................................    8     1    JCK-3
....................uNuggaucuuguuugacagca...................................................................................................................................................................................................    1     1    JCK-1
....................ucuggaucuuguuugacagca...................................................................................................................................................................................................    1097  0    JCK-2
....................ucuggaucuuguuugCcagca...................................................................................................................................................................................................    2     1    LT-3
....................ucuggaucuuguuugacagNa...................................................................................................................................................................................................    2     1    JCK-1
....................ucuggaucuugGuugacagca...................................................................................................................................................................................................    2     1    LT-1
....................ucuggaucuuguuugacagca...................................................................................................................................................................................................    832   0    JCK-3
....................uUuggaucuuguuugacagca...................................................................................................................................................................................................    12    1    JCK-2
....................ucuggaucuuguuugacagca...................................................................................................................................................................................................    195   0    LT-2
....................ucuggaucuuguuugacagcU...................................................................................................................................................................................................    6     1    LT-1
....................ucuggaucuuguuugacagca...................................................................................................................................................................................................    299   0    LT-1
....................ucuggauAuuguuugacagca...................................................................................................................................................................................................    1     1    JCK-2
....................ucuggaucuuguuugacGgca...................................................................................................................................................................................................    2     1    JCK-1
....................ucuggaucuuguuugacagUa...................................................................................................................................................................................................    1     1    JCK-2
....................ucuggaucuuguuugUcagca...................................................................................................................................................................................................    1     1    JCK-3
....................ucuggaucuuguuugacagcU...................................................................................................................................................................................................    9     1    LCK-3
....................ucuggCucuuguuugacagca...................................................................................................................................................................................................    1     1    JCK-2
....................ucuggaucuuguuugacagca...................................................................................................................................................................................................    12    0    JT-3
....................ucuggaucuuguuugacaUca...................................................................................................................................................................................................    3     1    JCK-3
....................ucuggaucCuguuugacagca...................................................................................................................................................................................................    1     1    JCK-2
....................ucuggaucuuguuugacagca...................................................................................................................................................................................................    9     0    LCK-1
....................ucuggaucuuguuugCcagca...................................................................................................................................................................................................    1     1    JCK-1
....................ucuggaucuCguuugacagca...................................................................................................................................................................................................    1     1    LT-3
....................ucuggaucuuguuugacagcU...................................................................................................................................................................................................    7     1    LT-3
....................ucuggaucuuguuugacagca...................................................................................................................................................................................................    267   0    LT-3
....................ucCggaucuuguuugacagca...................................................................................................................................................................................................    1     1    JCK-2
....................ucuggaucuuguuugGcagca...................................................................................................................................................................................................    1     1    LT-2
....................ucuggaucuuguuugacagca...................................................................................................................................................................................................    29    0    JT-1
....................ucuggaucuuguuugacagcC...................................................................................................................................................................................................    1     1    LCK-3
....................ucuggaucuugGuugacagca...................................................................................................................................................................................................    1     1    JCK-1
....................ucCggaucuuguuugacagca...................................................................................................................................................................................................    1     1    JCK-1
....................ucuggaucuuguuugacagcU...................................................................................................................................................................................................    3     1    JCK-2
....................ucuggaucuuguuugacagcN...................................................................................................................................................................................................    1     1    JCK-1
....................ucuggaucuuguuugUcagca...................................................................................................................................................................................................    1     1    JCK-2
....................ucuggaucuuguuugGcagca...................................................................................................................................................................................................    1     1    LCK-3
....................ucuggauUuuguuugacagca...................................................................................................................................................................................................    1     1    JCK-2
....................ucuggaucuuguuugacagca...................................................................................................................................................................................................    11    0    JT-2
....................ucuNgaucuuguuugacagca...................................................................................................................................................................................................    1     1    JCK-1
....................ucuggaucuuguuugacagca...................................................................................................................................................................................................    145   0    LCK-3
....................ucuggaucuuguuugacagcac..................................................................................................................................................................................................    27    0    LT-1
....................ucuggaucuuguuugacagcaU..................................................................................................................................................................................................    2     1    LT-2
....................ucuggaucuuguuugCcagcac..................................................................................................................................................................................................    1     1    LT-2
....................ucuggaucuuguuugacagcaU..................................................................................................................................................................................................    1     1    LT-1
....................ucuggaucuuguuugacagcac..................................................................................................................................................................................................    19    0    LCK-3
....................ucuggaucuuguuugacagcac..................................................................................................................................................................................................    50    0    JCK-2
....................ucuggaucuuguuugacagcac..................................................................................................................................................................................................    34    0    JCK-1
....................ucuggaucuuguuugacagcac..................................................................................................................................................................................................    24    0    LT-3
```

Mature **Star**

uccaucuuguucguuuacccucuggaucuuguuugacagcacucucuuauauuuugccauucaucuuuauaugccaaauggcgaugaugaugaugguggga gugcuaucgacaugauccagaaggugcagucgaauaagauacagagggaccacccacaguaccuacucucguuacaaucguauucuucuccucacuuauuauuauuauuauuauuauuccgaucauuuucuuuuaaucuauugaag

....................ucuggaucuuguuugacagcac	17	0	LT-2
....................ucuggaucuuguuugacagcac	22	0	JCK-3
....................ucuggaucuuguuugacagcacu	2	0	JCK-3
....................ucuggaucuuguuugacagcacu	5	0	JCK-2
....................ucuggaucuuguuugacagcacu	2	0	JCK-1
....................ucuggaucuuguuugacagcacu	3	0	LT-1
....................ucuggaucuuguuugacagcaUu	1	1	LT-3
....................ucuggaucuuguuugacagcacuc	2	0	LT-3
....................ucuggaucuuguuugacagcacuc	1	0	JCK-1
....................ucuggaucuuguuugacagcacuc	1	0	JCK-3
....................ucuggaucuuguuugacagcacuc	1	0	JCK-2
....................ucuggaucuuguuugacagcacucu	2	0	LT-1
.....................cuggaucuuguuugacagc	1	0	JCK-1
.....................cuggaucuuguuugacagc	7	0	JCK-2
.....................cuggaucuuguuugacagc	13	0	LT-2
.....................cuggaucuuguuugacagc	5	0	LCK-3
.....................cuggaucuuguuugacagc	1	0	LT-3
.....................cuggaucuuguuugacagc	5	0	LT-1
.....................cuggaucuuguuugacagc	2	0	JCK-3
.....................cuggaucuuguuugacagca	12	0	LCK-3
.....................cuggaucuuguuugacagca	15	0	LT-3
.....................cuggaucuuguuugacagcU	5	1	LT-2
.....................cuggaucuuguuugCcagca	1	1	JCK-1
.....................cuggaucuuguuugacagca	51	0	JCK-3
.....................cuggaucuuguuugacagcU	1	1	LT-3
.....................cuggaucuuguuugacagca	11	0	LT-2
.....................cuggaucuuguuugacagcU	4	1	JT-3
.....................cuggaucuuguuugacagcU	1	1	JCK-3
.....................cuggaucuuguuugacagca	61	0	JCK-2
.....................cuggaucuuguuugacagca	9	0	LT-1
.....................cuggaucuuguuugacagcU	4	1	JT-2
.....................cuggaucuuguuugacagcU	1	1	JCK-2
.....................cuggaucuuguuugacagca	58	0	JCK-1
.....................cuggaucuuguuugacagcac	3	0	LT-3
.....................cuggaucuuguuugacagcac	4	0	LT-2
.....................cuggaucuuguuugacagcac	1	0	LCK-3
.....................cuggaucuuguuugacagcacu	13	0	LT-3
.....................cuggaucuuguuugacagcacu	22	0	JCK-3
.....................cuggaucuuguuugacagcaUu	2	1	JCK-3
.....................cuggaucuuguuugacagcacu	7	0	LT-2
.....................cuggaucuuguuugacagcaUu	1	1	JCK-2
.....................cuggaucuuguuugacagcacu	17	0	JCK-1
.....................cuggaucuuguuugacagcacu	4	0	LT-1
.....................cuggaucuuguuugacagcacu	15	0	LCK-3
.....................cuggaucuuguuugacagcacu	46	0	JCK-2
.....................cuggaucuuguuugacagcacuc	3	0	JCK-3
.....................cuggaucuuguuugacagcacuc	1	0	JCK-2
.....................cuggaucuuguuugacagcacuc	1	0	JCK-1
.....................cuggaucuuguuugacagcacucu	3	0	JCK-2
.....................cuggaucuuguuugacagcacucuc	1	0	JCK-1
.....................cuggaucuuguuugacagcacucuc	3	0	JCK-2
......................uggaucuuAuuugacagc	3	1	LT-2
......................uggaucuuAuuugacagc	3	1	LT-1
......................ugAaucuuguuugacagc	3	1	LCK-3
......................uggCucuuguuugacagc	2	1	JCK-2
......................uAgaucuuguuugacagc	4	1	LT-3
......................uggaucuuguuugGcagc	2	1	LCK-1
......................uggaucuuguuugacagc	520	0	JT-1
......................uggCucuuguuugacagc	2	1	JT-2
......................uggaucuuguGugacagc	1	1	LT-2
......................uggaucuugCuugacagc	1	1	LT-1
......................uggCucuuguuugacagc	3	1	JT-1
......................uggaucuuguuugCcagc	2	1	LCK-1
......................ugAaucuuguuugacagc	2	1	LCK-1
......................uggaucuuAuuugacagc	1	1	LCK-2
......................uggaucuuguuugacagU	13	1	LCK-3
......................uggaucuuguuugacagA	3	1	JCK-1
......................uNgaucuuguuugacagc	1	1	LCK-3
......................uggaucuuguCugacagc	2	1	JCK-2
......................uggaucuuguuuAacagc	1	1	LCK-3

Mature **Star**

uccaucuuguucguuuacccucuggaucuuguuugacagcacucucuuauauuuugccauucaucuuuauaugccaaauggcgaugaugaugauggugggagugcuaucgacaugauccagaaggugcagucgaauaagauacagagggaccacccacaguaccuacucucguuacaaucguauucuucuccucacuuauuauuauuauuauuauuauuauuccgaucauuuucuuuuaaucuauugaag

uggaucuuguuugacagc	2286	0	LT-1
uggaucuuguuuUacagc	1	1	JT-2
uggaucuugCuugacagc	1	1	JCK-1
uggaucuuguuugacagG	1	1	JCK-1
uggGucuuguuugacagc	1	1	LCK-3
uggCucuuguuugacagc	7	1	LT-3
uggaucuuguuugaNagc	11	1	LCK-1
ugNaucuuguuugacagc	7	1	LT-2
uggaucuuguuNgacagc	1	1	JCK-2
uggaucuuguuugCcagc	4	1	LT-3
uggaucuuguuugCcagc	4	1	JCK-2
Cggaucuuguuugacagc	1	1	LT-3
uggaucuuguuugCcagc	3	1	JCK-1
uggaucuuguuugacCgc	6	1	LT-2
uggaucuuguuuNacagc	3	1	LCK-1
ugAaucuuguuugacagc	1	1	LT-1
uggaucuuguuuAacagc	1	1	LCK-2
uggaucuuguuugCcagc	2	1	LCK-2
uggaucuuguuugacagU	1	1	JCK-3
uggNucuuguuugacagc	1	1	JT-2
uUgaucuuguuugacagc	1	1	LCK-3
uNgaucuuguuugacagc	1	1	JCK-3
uNgaucuuguuugacagc	2	1	LCK-1
uggaucuuguuugacagU	1	1	JCK-1
ugAaucuuguuugacagc	1	1	JCK-2
uggaucuAguuugacagc	1	1	LT-1
ugUaucuuguuugacagc	1	1	LCK-3
uggauNuuguuugacagc	1	1	LCK-1
uggaucuuguuugacCgc	2	1	LCK-3
uggaucuuguuugacaCc	2	1	JCK-3
uggaucuuguuugacagU	12	1	LT-3
uUgaucuuguuugacagc	1	1	JCK-2
uggaucuuguuugacagc	6351	0	LCK-3
uAgaucuuguuugacagc	4	1	LCK-3
uggaucuuguuugacCgc	2	1	JCK-2
uggaucuuguuugacagc	419	0	JT-3
uggaucuugCuugacagc	1	1	JCK-2
uggCucuuguuugacagc	3	1	LCK-2
uggaucuuguuugacagA	6	1	LT-3
uggaucuuguuugacCgc	2	1	LCK-2
uggGucuuguuugacagc	1	1	LT-2
uggaucuuguuugacagU	3	1	JCK-2
uggaucuuguuugCcagc	24	1	LT-2
uggCucuuguuugacagc	2	1	LCK-1
uggaucuuguuugaGagc	4	1	LCK-1
uAgaucuuguuugacagc	5	1	LT-2
uggCucuuguuugacagc	5	1	LT-1
uggaucuuguuugCcagc	15	1	LCK-3
uggCucuuguuugacagc	10	1	LCK-3
uggaucuuguuugacCgc	2	1	LT-1
uNgaucuuguuugacagc	1	1	JT-1
uCgaucuuguuugacagc	1	1	LT-1
uggaucCuguuugacagc	1	1	LT-3
uggNucuuguuugacagc	1	1	JCK-1
ugUaucuuguuugacagc	1	1	JCK-1
ugNaucuuguuugacagc	1	1	LCK-1
uNgaucuuguuugacagc	1	1	JCK-1
ugAaucuuguuugacagc	1	1	LT-3
uggaucuuguuNgacagc	1	1	JT-1
uggaucuuguuNgacagc	1	1	JCK-1
uggaucuuguuugacagc	353	0	JT-2
uggaucuuguuugNcagc	6	1	LCK-1
uggaucuCguuugacagc	1	1	LT-1
uggaucuuguuugacagc	1255	0	JCK-3
uNgaucuuguuugacagc	3	1	JCK-2
uggaucuuguuugacagA	7	1	LT-2
uggaucuuguuugacagc	1447	0	LCK-1
uggaucuuguuugCcagc	6	1	LT-1
uUgaucuuguuugacagc	1	1	LT-3
uggaucuuguuugacagc	1810	0	JCK-2

Mature **Star**

uccaucuuguucguuuacccuc**uggaucuuguuugacagcacu**cucuuauauuuugccauucaucuuuauaugccaaauggcgaugaugaugaugguggga**gugcuaucgacaugauccaga**aggugcagucgaauaagauacagagggaccacccacaguaccuacucucguuacaaucguauucuucuccucacuuauuauuauuauuauuauuauuauuccgaucauuuucuuuuaaucuauugaag

Cggaucuuguuugacagc	1	1	LT-2
uggaucuuguuugacagU	9	1	LT-1
uggaucuuguuugacagA	1	1	JCK-3
uggCucuuguuugacagc	53	1	LT-2
uggaucuuguuugacCgc	1	1	JT-1
uggaucuuguuugacagA	1	1	LCK-1
uggaucuuguuugacagc	1573	0	LCK-2
uAgaucuuguuugacagc	1	1	LCK-2
uggaucuuguuugacagU	21	1	LT-2
uggCucuuguuugacagc	1	1	JCK-3
uggCucuuguuugacagc	2	1	JT-3
ugUaucuuguuugacagc	1	1	LT-2
uNgaucuuguuugacagc	1	1	LT-3
uggaucuuguuugacagc	1080	0	JCK-1
ugAaucuuguuugacagc	3	1	LT-2
uggaucuuguuugacagc	2328	0	LT-3
uggaucuuguuugCcagc	1	1	JCK-3
uUgaucuuguuugacagc	1	1	LT-2
uggCucuuguuugacagc	1	1	JCK-1
uggaucuuguuugacagc	5296	0	LT-2
uAgaucuuguuugacagc	1	1	LT-1
uggaucuuAuuugacagc	1	1	JCK-1
uggaucuuguuugacGgc	1	1	LT-2
uggaucuuguuugacagA	4	1	LCK-3
uggaucuuAuuugacagc	1	1	LT-3
uggaucuuguuugacagUa	11	1	JCK-3
uggaucuuAuuugacagca	1	1	LCK-2
ugNaucuuguuugacagca	9	1	LT-2
uggaucuuguuugacagcG	15	1	LT-2
uggaucuuguuugacCgca	1	1	JT-1
uggaucuuguuugacagca	3565	0	LCK-1
uggaucuuguuugacagca	1294	0	JT-3
uAgaucuuguuugacagca	1	1	LCK-2
uggCucuuguuugacagca	7	1	LCK-2
uggaucuugCuugacagca	1	1	JT-1
ugUaucuuguuugacagca	1	1	LCK-2
uggaucuuguuugacCgca	2	1	JCK-2
uggaucuuguuugCcagca	3	1	LCK-1
uggaucuuguuugCcagca	11	1	LT-2
uggaucuuguuugacagcC	14	1	LT-1
uggaucuuguuugNcagca	9	1	LCK-1
uAgaucuuguuugacagca	1	1	LT-3
uggaucuuguuugacagcU	1354	1	LT-1
uggaucuuguuugacagAa	1	1	LT-3
uggaucuuguuugacagca	1862	0	JCK-2
uggaucuuguuugacagca	1154	0	JCK-1
uggaucuuguuugacagca	858	0	LT-1
uggaucuuguuugacCgca	1	1	JCK-3
uggaucuuguuuNacagca	2	1	LCK-1
ugNaucuuguuugacagca	5	1	LCK-1
uggaucGuguuugacagca	1	1	LCK-2
uggaucuuguuugacagUa	24	1	JCK-2
uggaucuuguuugacagcG	2	1	JCK-2
uggaucuuguuuUacagca	1	1	LCK-3
uggaucuuguuugacagcC	7	1	LCK-1
uggaucuuguuugacCgca	2	1	LCK-1
uggaucuuguuugacagcU	718	1	JCK-1
uNgaucuuguuugacagca	3	1	JT-1
uggaucuuguuugaAagca	1	1	LCK-2
uggaucuuguuugCcagca	6	1	LCK-2
uggaucuuguuugacagcC	5	1	JCK-2
uggaucuuguuugacagca	1105	0	JCK-3
uggaucuuguuugacagcU	835	1	JCK-3
uggCucuuguuugacagca	2	1	JCK-1
uggCucuuguuugacagca	2	1	JT-3
uggaucuCguuugacagca	1	1	LT-1
uggaucuuguuugacCgca	2	1	JT-3
uNgaucuuguuugacagca	1	1	JCK-3
uggaucuuUuuugacagca	1	1	LCK-2
uggaucuuguuugacagca	2333	0	LT-2

Mature Star

uccaucuuguucguuuacccucuggaucuuguuugacagcacucucuuauauuuugccauucaucuuuauaugccaaauggcgaugaugaugauggugggagugcuaucgacaugauccagaaggugcagucgaauaagauacagagggaccacccacaguaccuacucucguuacaaucguauucuucuccucacuuauuauuauuauuauuauuauuccgaucauuucuuuuaaucuauugaag			
......................uggaucuuguuugacagcG...	12	1	LT-1
......................uggaucuuguuugacagcU...	1214	1	JCK-2
......................uggaucuuguuugacagcG...	2	1	JCK-3
......................uggaucuuguuugaAagca...	1	1	LCK-3
......................uggaucuuguuugacagcU...	381	1	JT-3
......................uggaucuuguuugacagcU...	735	1	JT-1
......................uggaucuuguuugacagcU...	279	1	JT-2
......................uggaucuuguuugCcagca...	1	1	LT-1
......................uggaucuuguuugaGagca...	4	1	LCK-1
......................uggaucuuguuugCcagca...	2	1	JCK-1
......................uNgaucuuguuugacagca...	2	1	JCK-2
......................uggaucuCguuugacagca...	1	1	LCK-2
......................uggCucuuguuugacagca...	4	1	JCK-2
......................uggaucuuguuugacagUa...	1	1	LCK-2
......................uggCucuuguuugacagca...	3	1	JT-2
......................uggCucuuguuugacagca...	2	1	LT-1
......................uggaucuuguuugacagca...	3109	0	LCK-3
......................uggaucuuguuugacagUa...	3	1	LT-1
......................ugAaucuuguuugacagca...	1	1	JCK-3
......................uUgaucuuguuugacagca...	1	1	LT-3
......................ugAaucuuguuugacagca...	1	1	JT-1
......................uggauNuuguuugacagca...	10	1	LCK-1
......................uggauUuuguuugacagca...	1	1	LT-2
......................uggaucuuguuugacagcC...	2	1	JCK-1
......................uggaucuuguuugacagUa...	29	1	LT-2
......................ugAaucuuguuugacagca...	1	1	LT-3
......................uggaucCuguuugacagca...	1	1	LCK-1
......................uggaucuuguuugacagcU...	2784	1	LT-2
......................uggaucuuguuugacagcU...	2081	1	LT-3
......................uggaucuuguuugacagUa...	10	1	JT-3
......................uggaucuuguuGgacagca...	9	1	LCK-3
......................uggaucuuguuugacagcC...	20	1	LT-2
......................uggaucuuguuugGcagca...	1	1	LT-2
......................uggaCcuuguuugacagca...	1	1	LT-2
......................uggaucuuguuugacagcG...	2	1	JT-2
......................uggGucuuguuugacagca...	1	1	JCK-1
......................uggaucuCguuugacagca...	1	1	JT-1
......................uggNucuuguuugacagca...	1	1	LCK-1
......................uggaucuuguuugacCgca...	1	1	LT-2
......................uggaucuuguuugacagcU...	1503	1	LCK-2
......................uggaucuuguuugacagUa...	11	1	JT-2
......................uggaucuuguuugCcagca...	2	1	LT-3
......................uggCucuuguuugacagca...	5	1	LCK-1
......................uggaucuuguuugacagUa...	11	1	LT-3
......................uggaucuuguuNgacagca...	1	1	JT-2
......................uggaucuuguuugacCgca...	3	1	LCK-3
......................uggaucuuguuNgacagca...	2	1	JT-1
......................uggaucuuguuugacagca...	980	0	JT-2
......................uggaucCuguuugacagca...	1	1	LT-3
......................uggaucuuguuugCcagca...	2	1	LCK-3
......................uggCucuuguuugacagca...	12	1	LT-2
......................uggaucuCguuugacagca...	1	1	LT-2
......................uggaucuuguuugacCgca...	2	1	LT-3
......................uUgaucuuguuugacagca...	5	1	JT-3
......................uggaucuugNuugacagca...	1	1	LCK-1
......................uggauAuuguuugacagca...	1	1	JCK-3
......................uggaucuuguuugacagUa...	18	1	LCK-3
......................uggaucuuguuugacagcC...	3	1	JT-1
......................uggaucuuguuugacagcC...	21	1	LCK-3
......................uggCucuuguuugacagca...	2	1	LT-3
......................uggaucuuguuugacagca...	1426	0	JT-1
......................uggaucuuguuugaNagca...	11	1	LCK-1
......................uggaucuuguuugacagcC...	8	1	LCK-2
......................uggGucuuguuugacagca...	1	1	LCK-2
......................uggaucuuguuugacCgca...	1	1	JCK-1
......................uggaucuuguuugacagcG...	11	1	LCK-3
......................uggaucuuguuugacagUa...	12	1	JCK-1
......................uggaucuuAuuugacagca...	1	1	LCK-3
......................Cggaucuuguuugacagca...	1	1	LCK-3
......................uggCucuuguuugacagca...	5	1	JT-1

```
                      Mature                                                                              Star
uccaucuuguucguuuacccucuggaucuuguuugacagcacucucuuauauuuugccauucaucuuuauaugccaaauggcgaugaugaugaugguggga gugcuaucgacaugauccagaaggugcagucgaauaagauacagagggaccacccacaguaccuacucucguuacaaucguauucuucuccucacuuauuauuauuauuauuauuauuccgaucauuuucuuuuaaucuauugaag
......................uggaucuuguuugacagca.........................................................................................................................................................................................................   3633   0   LCK-2
......................uggaucuuguuugGcagca.........................................................................................................................................................................................................   2      1   LCK-1
......................uggaucuuguuugacagca.........................................................................................................................................................................................................   1490   0   LT-3
......................uggaucuuguuugCcagca.........................................................................................................................................................................................................   5      1   JCK-2
......................uggaucuuguuugacagcC.........................................................................................................................................................................................................   21     1   LT-3
......................uNgaucuuguuugacagca.........................................................................................................................................................................................................   1      1   LCK-3
......................uggaucuuguuugacagcC.........................................................................................................................................................................................................   6      1   JCK-3
......................uggCucuuguuugacagca.........................................................................................................................................................................................................   3      1   JCK-3
......................uAgaucuuguuugacagca.........................................................................................................................................................................................................   3      1   LCK-3
......................uggaucuuguuNgacagca.........................................................................................................................................................................................................   5      1   JCK-1
......................uggaucuuguuugacagcG.........................................................................................................................................................................................................   11     1   LT-3
......................uggCucuuguuugacagca.........................................................................................................................................................................................................   4      1   LCK-3
......................uggaucuuguuugacagcG.........................................................................................................................................................................................................   6      1   JCK-1
......................uggaucCuguuugacagca.........................................................................................................................................................................................................   1      1   LT-2
......................uAgaucuuguuugacagca.........................................................................................................................................................................................................   1      1   LT-2
......................uggaucuuguuugGcagca.........................................................................................................................................................................................................   1      1   JT-2
......................uUgaucuuguuugacagca.........................................................................................................................................................................................................   5      1   JT-2
......................uggaucuuguuugacagcU.........................................................................................................................................................................................................   1372   1   LCK-1
......................uNgaucuuguuugacagca.........................................................................................................................................................................................................   2      1   JCK-1
......................uggaucuuguuugacagcU.........................................................................................................................................................................................................   4465   1   LCK-3
......................uAgaucuuguuugacagca.........................................................................................................................................................................................................   1      1   JT-1
......................uggaucGuguuugacagca.........................................................................................................................................................................................................   1      1   JT-3
......................uggaucuuguuugacagcaU........................................................................................................................................................................................................   22     1   LT-1
......................uNgaucuuguuugacagcac........................................................................................................................................................................................................   1      1   JCK-1
......................uggaucuuguuugacagcaN........................................................................................................................................................................................................   2      1   JCK-1
......................uggaucuuguuugacagcaU........................................................................................................................................................................................................   26     1   LT-3
......................uggaucuuguuugCcagcac........................................................................................................................................................................................................   2      1   JT-1
......................uggaucuuguuugacagcac........................................................................................................................................................................................................   899    0   JCK-2
......................uggaucuuguuugacagcCc........................................................................................................................................................................................................   1      1   JT-1
......................uggaucuuguuugacagcUc........................................................................................................................................................................................................   6      1   JCK-1
......................uggaucuuguuugGcagcac........................................................................................................................................................................................................   1      1   LCK-1
......................uggaucuuguuugacCgcac........................................................................................................................................................................................................   1      1   LT-2
......................uggaucuuguuugacagcac........................................................................................................................................................................................................   586    0   JCK-1
......................Aggaucuuguuugacagcac........................................................................................................................................................................................................   1      1   LCK-3
......................uggaucuuguuugCcagcac........................................................................................................................................................................................................   1      1   LT-3
......................uggaucuuguuugCcagcac........................................................................................................................................................................................................   4      1   LT-2
......................uggCucuuguuugacagcac........................................................................................................................................................................................................   1      1   LCK-2
......................uggCucuuguuugacagcac........................................................................................................................................................................................................   1      1   LCK-1
......................uggaucuuguCugacagcac........................................................................................................................................................................................................   1      1   JCK-2
......................uggaucuuguuugacagcUc........................................................................................................................................................................................................   36     1   LT-1
......................uggaCcuuguuugacagcac........................................................................................................................................................................................................   1      1   JCK-2
......................uggaucuugCuugacagcac........................................................................................................................................................................................................   1      1   LT-2
......................uggaucuuguuugacagcac........................................................................................................................................................................................................   715    0   LT-3
......................uggaucuuguuugacCgcac........................................................................................................................................................................................................   2      1   JCK-2
......................uggaucuuguuugacagcac........................................................................................................................................................................................................   170    0   JT-2
......................uAgaucuuguuugacagcac........................................................................................................................................................................................................   1      1   LCK-3
......................ugNaucuuguuugacagcac........................................................................................................................................................................................................   3      1   LT-2
......................uggCucuuguuugacagcac........................................................................................................................................................................................................   1      1   JCK-1
......................uggaucuuguuugacagcUc........................................................................................................................................................................................................   26     1   LCK-1
......................uAgaucuuguuugacagcac........................................................................................................................................................................................................   4      1   LT-2
......................uggaucuuguuugCcagcac........................................................................................................................................................................................................   1      1   LCK-3
......................uggaucuuguuugacagcaA........................................................................................................................................................................................................   4      1   JCK-3
......................uggCucuuguuugacagcac........................................................................................................................................................................................................   9      1   LT-2
......................uggaucuuguuugCcagcac........................................................................................................................................................................................................   1      1   LT-1
......................uAgaucuuguuugacagcac........................................................................................................................................................................................................   1      1   LCK-1
......................uggaucuuguuugacagcUc........................................................................................................................................................................................................   31     1   LCK-2
......................uggaucuuguuugCcagcac........................................................................................................................................................................................................   1      1   JT-2
......................uggaucuuguuugacagcac........................................................................................................................................................................................................   216    0   JT-1
......................uggaucuuguuugacagcac........................................................................................................................................................................................................   982    0   LT-2
......................uggaucuuguuugacagUac........................................................................................................................................................................................................   1      1   JCK-3
......................uggaucuuguuugCcagcac........................................................................................................................................................................................................   3      1   JCK-2
......................uggCucuuguuugacagcac........................................................................................................................................................................................................   5      1   LCK-3
......................uggaucuuguuugacagcaU........................................................................................................................................................................................................   8      1   JCK-1
......................uggaucuuguuugacGgcac........................................................................................................................................................................................................   1      1   LT-2
......................uggaucuuguuugacagcNc........................................................................................................................................................................................................   1      1   JCK-1
......................uggaucuuguuugacagcaA........................................................................................................................................................................................................   3      1   LT-3
......................uggaucuuguuugGcagcac........................................................................................................................................................................................................   1      1   LT-3
......................uNgaucuuguuugacagcac........................................................................................................................................................................................................   1      1   LCK-3
......................uggaucuuguuugacagcaU........................................................................................................................................................................................................   26     1   LT-2
......................uggaucuuUuuugacagcac........................................................................................................................................................................................................   1      1   LT-3
```

Mature **Star**

uccaucuuguucguuuacccucuggaucuuguuugacagcacucucuuauauuuugccauucaucuuuauaugccaaauggcgaugaugaugauggugggagugcuaucgacaugauccagaaggugcagucgaauaagauacagagggaccacccacaguaccuacucucguuacaaucguauucuucuccucacuuauuauuauuauuauuauuauuccgaucauuuucuuuuaaucuauugaag			
......................uggaucuuguuugacagcac...	545	0	LCK-1
......................uggCucuuguuugacagcac...	2	1	JT-3
......................uggaucuuguuugacagcaA...	1	1	JCK-2
......................uggaCcuuguuugacagcac...	1	1	JCK-3
......................uggaucuuguuugacagcGc...	1	1	LCK-3
......................uggaucuuguuugacagcCc...	1	1	LT-2
......................uggaucuuguuugacagcaA...	3	1	JCK-1
......................uggaucuuguuugacagcaU...	26	1	JCK-3
......................uggaucuuguuugacagcaA...	1	1	JT-3
......................uggaucuuguuugacagcaG...	1	1	LT-1
......................ugAaucuuguuugacagcac...	1	1	JCK-1
......................uggaucuuCuuugacagcac...	1	1	LCK-3
......................uggaucuuguuugNcagcac...	2	1	LCK-1
......................uggaucuuguuugaNagcac...	2	1	LCK-1
......................uggauNuuguuugacagcac...	1	1	LCK-1
......................uggaucuuguuuAacagcac...	1	1	LCK-2
......................uggaucuuguuugCcagcac...	1	1	JCK-3
......................uggCucuuguuugacagcac...	2	1	LT-3
......................uggaucuuguuNgacagcac...	1	1	JT-2
......................uggaucuuguuugacagcac...	249	0	JT-3
......................uAgaucuuguuugacagcac...	1	1	LT-1
......................uggaCcuuguuugacagcac...	1	1	JT-1
......................uggaucuuguuugacagcUc...	51	1	LT-3
......................uggGucuuguuugacagcac...	2	1	LT-2
......................uggCucuuguuugacagcac...	1	1	LT-1
......................uggaucuuguuugCcagcac...	1	1	JCK-1
......................uggaucuuguuugacagcUc...	77	1	LT-2
......................uggaucuuguuugacagcaU...	66	1	LCK-3
......................uggaucuuguuugacagcUc...	7	1	JCK-3
......................uggCucuuguuugacagcac...	2	1	JCK-2
......................uggaucuuguuugacagcUc...	63	1	LCK-3
......................uggaucuuguuugacagcac...	666	0	LT-1
......................uggaucuuguuugacagcaU...	46	1	JCK-2
......................uggaucuuguuugacagcac...	687	0	JCK-3
......................uggaucuuguuugacagcCc...	2	1	LT-1
......................uggaucuuguuugacagcaU...	16	1	JT-1
......................uggaucuuguuugacCgcac...	3	1	LT-3
......................uggaucuuguuugacagcac...	1318	0	LCK-3
......................uggaucuuguuugacagcUc...	15	1	JCK-2
......................uggaucuuguuugacagcaA...	1	1	LCK-2
......................uggaucuuguuugacagcac...	525	0	LCK-2
......................uggaucuuguuugacagUacu..	18	1	JCK-1
......................Cggaucuuguuugacagcacu..	3	1	LT-1
......................uggaucuCguuugacagcacu..	4	1	JCK-3
......................uggauNuuguuugacagcacu..	1	1	JCK-2
......................ugNaucuuguuugacagcacu..	1	1	LCK-1
......................uggNucuuguuugacagcacu..	3	1	JCK-3
......................uggaucuuguuugacagcacG..	2	1	LCK-1
......................uggaucuuguuuAacagcacu..	1	1	LCK-3
......................uggaucuuguuuAacagcacu..	1	1	LCK-1
......................uggaucuuguuugacagcaUu..	2361	1	JCK-2
......................uggaucuuguuugacagcaUu..	22	1	JT-2
......................uggaucuuguuugacagcGcu..	2	1	JCK-2
......................ugNaucuuguuugacagcacu..	65	1	LT-2
......................uggaucuuguuugacagAacu..	1	1	JCK-1
......................uggaCcuuguuugacagcacu..	14	1	JCK-2
......................uggaucuuguGugacagcacu..	2	1	LT-2
......................uAgaucuuguuugacagcacu..	8	1	JCK-2
......................uggaucuuguCugacagcacu..	1	1	LT-3
......................uggaucuuguuugacagcNcu..	1	1	LCK-1
......................uggaucuuguuugacagcCcu..	74	1	JCK-2
......................uggaucuuAuuugacagcacu..	1	1	LCK-1
......................uggCucuuguuugacagcacu..	200	1	LT-2
......................uggaucuuguuuAacagcacu..	9	1	JCK-2
......................uggaucCuguuugacagcacu..	2	1	LT-3
......................uggaucuuguuugacagcacu..	5029	0	LCK-2
......................uggaucuuguuuCacagcacu..	1	1	JCK-2
......................uggaucuuguuugacagcaAu..	3	1	JCK-3
......................uggaucuuCuuugacagcacu..	1	1	JCK-1
......................uggaucuuguuugGcagcacu..	1	1	LT-2

Mature Star

uccaucuuguucguuuacccucuggaucuuguuugacagcacucucuuauauuuugccauucaucuuuauaugccaaauggcgaugaugaugaugguggga gugcuaucgacaugauccagaaggugcagucgaauaagauacagagggaccacccacaguaccuacucucguuacaaucguauucuucuccucacuuauuauuauuauuauuauuauuccgaucauuuucuuuuaaucuauugaag

uggaucuGguuugacagcacu	1	1	JCK-2
uggaucuuguuugacagcCcu	2	1	JT-1
uggCucuuguuugacagcacu	95	1	LT-3
uggaucuuguuugacagcaAu	3	1	JCK-1
uggaucuuguCugacagcacu	3	1	LT-1
uggaucuuguuuUacagcacu	2	1	LT-2
uggaucCuguuugacagcacu	8	1	JCK-3
uggaucuuguuugacagcCcu	16	1	LCK-3
uggaucuuguuugacagcacu	38814	0	JCK-1
uggaucuuguuGgacagcacu	1	1	LT-3
uggaucuuguuugacagGacu	2	1	JCK-2
ugUaucuuguuugacagcacu	1	1	LCK-2
uggaucGuguuugacagcacu	1	1	LT-1
uggaucuuguuugacagcaUu	36	1	LCK-1
uggaucuuguuugGcagcacu	2	1	LT-1
uggaCcuuguuugacagcacu	4	1	JCK-1
uggaucuuguuugacagcaNu	42	1	JCK-1
uggaucuuguuugacagcaUu	37	1	LCK-2
ugUaucuuguuugacagcacu	1	1	JT-1
uggaucuuguuugacagcacu	4848	0	LCK-1
uggaucuuguuugGcagcacu	3	1	JCK-3
uggaucuuguuuUacagcacu	2	1	JCK-3
uggaucuuguuGgacagcacu	1	1	JCK-2
uUgaucuuguuugacagcacu	10	1	JCK-3
uggaucuuguuugCcagcacu	80	1	LT-2
uggaucuuguuugCcagcacu	61	1	LT-1
uggaucuuAuuugacagcacu	4	1	LCK-3
uggaucCuguuugacagcacu	4	1	LT-1
uggaucuuguuugCcagcacu	2	1	JT-2
ugUaucuuguuugacagcacu	1	1	JCK-2
uggaucuuguCugacagcacu	6	1	JCK-2
uggaucuuguuugacagcUcu	1	1	LT-3
uggauUuuguuugacagcacu	1	1	LCK-3
Gggaucuuguuugacagcacu	1	1	JCK-2
ugAaucuuguuugacagcacu	6	1	LT-1
Cggaucuuguuugacagcacu	4	1	LT-2
uggaucuuguuugaNagcacu	17	1	LCK-1
uggaucuuguuugUcagcacu	2	1	JCK-2
uggaucuuguuNgacagcacu	121	1	JCK-1
uggaucuuAuuugacagcacu	6	1	LT-1
uggaucuuUuuugacagcacu	3	1	JCK-1
uNgaucuuguuugacagcacu	2	1	LT-3
uggauUuuguuugacagcacu	3	1	JCK-1
uggaucuuguuugacagcacG	16	1	JCK-2
uggaucuuguuugGcagcacu	3	1	JCK-1
uggaucuuguuNgacagcacu	1	1	JT-1
uggauAuuguuugacagcacu	1	1	JCK-3
uggaucuuguuugacCgcacu	5	1	LCK-2
uggaucuuguuugacagcaUu	21	1	JT-3
uUgaucuuguuugacagcacu	1	1	JT-3
uggaucuuguuugacagUacu	13	1	JCK-3
uggauNuuguuugacagcacu	3	1	LCK-1
uggaucuuguuugacGgcacu	9	1	JCK-3
uggaucuuguuuUacagcacu	6	1	JCK-2
uggaucuuguuugaAagcacu	3	1	LT-1
uggaucuuguuugacagNacu	3	1	JCK-3
uggaucuuguuugCcagcacu	1	1	JT-3
uggaucuuguuugacaCcacu	1	1	LT-3
ugUaucuuguuugacagcacu	2	1	LT-2
uggaucuuguuugacagcGcu	1	1	JT-3
uggauUuuguuugacagcacu	11	1	JCK-2
uggaucuuguuugacagcaAu	10	1	JCK-2
uggaucuCguuugacagcacu	10	1	JCK-2
uggaucuugCuugacagcacu	2	1	JCK-1
uggaucuuUuuugacagcacu	1	1	LCK-3
uggNucuuguuugacagcacu	22	1	JCK-1
uggaucuuguuugacGgcacu	3	1	LT-3
uggaucuuUuuugacagcacu	3	1	LT-2
uggaucuCguuugacagcacu	1	1	LCK-1
uggaucuuguuugacagcacu	48441	0	JCK-3

```
                      Mature                                                                                  Star
uccaucuuguucguuuacccucuggaucuuguuugacagcacucucuuauauuuugccauucaucuuuauaugccaaauggcgaugaugaugaugguggga gugcuaucgacaugauccagaaggugcagucgaauaagauacagagggaccacccacaguaccuacucucguuacaaucguauucuucuccucacuuauuauuauuauuauuauuauuccgaucauuuucuuuuaaucuauugaag
......................uggaucuuguuugacagcacu............................................................................................................................................................................................................ 979 0 JT-3
......................uggaucuuAuuugacagcacu............................................................................................................................................................................................................ 20 1 JCK-2
......................uggaucuugCuugacagcacu............................................................................................................................................................................................................ 6 1 JCK-2
......................uggaucuuguuugacaCcacu............................................................................................................................................................................................................ 1 1 JT-1
......................uggaucuCguuugacagcacu............................................................................................................................................................................................................ 3 1 LT-3
......................uggaucuuguuugacagcaAu............................................................................................................................................................................................................ 1 1 LCK-3
......................uggaucuuguuugacCgcacu............................................................................................................................................................................................................ 11 1 LT-3
......................uggaucuugAuugacagcacu............................................................................................................................................................................................................ 1 1 JCK-2
......................uggaucuuguuugacagcCcu............................................................................................................................................................................................................ 53 1 JCK-1
......................uggaucuuguuugaGagcacu............................................................................................................................................................................................................ 1 1 JT-1
......................uggaucuuguuugacagcacG............................................................................................................................................................................................................ 10 1 LT-1
......................uggaucuuguuuCacagcacu............................................................................................................................................................................................................ 1 1 JCK-3
......................uggaucuuguuugacagcaUu............................................................................................................................................................................................................ 102 1 LCK-3
......................Cggaucuuguuugacagcacu............................................................................................................................................................................................................ 9 1 LT-3
......................uNgaucuuguuugacagcacu............................................................................................................................................................................................................ 1 1 LCK-1
......................ugAaucuuguuugacagcacu............................................................................................................................................................................................................ 4 1 LCK-3
......................uggaucuuguuugacaCcacu............................................................................................................................................................................................................ 3 1 JCK-2
......................uggaucuuguuugCcagcacu............................................................................................................................................................................................................ 159 1 JCK-2
......................uggaucuugCuugacagcacu............................................................................................................................................................................................................ 4 1 LT-2
......................Cggaucuuguuugacagcacu............................................................................................................................................................................................................ 11 1 JCK-2
......................uggaucuuguuugacagcacu............................................................................................................................................................................................................ 20839 0 LT-2
......................uggaucuuguuugacagcaAu............................................................................................................................................................................................................ 4 1 JT-3
......................uNgaucuuguuugacagcacu............................................................................................................................................................................................................ 1 1 LCK-2
......................uggaucuuguuugacagcacG............................................................................................................................................................................................................ 7 1 LCK-3
......................uggaucuuNuuugacagcacu............................................................................................................................................................................................................ 1 1 JCK-3
......................ugUaucuuguuugacagcacu............................................................................................................................................................................................................ 2 1 JCK-1
......................uAgaucuuguuugacagcacu............................................................................................................................................................................................................ 4 1 LCK-2
......................uggaucuuguuugacCgcacu............................................................................................................................................................................................................ 28 1 JCK-3
......................uggaucuuguuugacagcGcu............................................................................................................................................................................................................ 3 1 LT-2
......................uggaucuugCuugacagcacu............................................................................................................................................................................................................ 4 1 LCK-3
......................uggCucuuguuugacagcacu............................................................................................................................................................................................................ 1 1 JT-3
......................uUgaucuuguuugacagcacu............................................................................................................................................................................................................ 2 1 LT-1
......................uggaucuuguuugacagcacG............................................................................................................................................................................................................ 3 1 LT-2
......................uggaucuuAuuugacagcacu............................................................................................................................................................................................................ 1 1 LCK-2
......................ugUaucuuguuugacagcacu............................................................................................................................................................................................................ 3 1 LT-3
......................uggaucuuguuuAacagcacu............................................................................................................................................................................................................ 1 1 JT-1
......................uggaucuuguuugacagcUcu............................................................................................................................................................................................................ 5 1 LT-1
......................uggaucuuguuNgacagcacu............................................................................................................................................................................................................ 1 1 JCK-2
......................ugAaucuuguuugacagcacu............................................................................................................................................................................................................ 10 1 LT-2
......................uggCucuuguuugacagcacu............................................................................................................................................................................................................ 39 1 LCK-3
......................uggaucuuguuugacagcacu............................................................................................................................................................................................................ 658 0 JT-2
......................uggaucuuguuugacGgcacu............................................................................................................................................................................................................ 10 1 JCK-2
......................uggaucuuAuuugacagcacu............................................................................................................................................................................................................ 6 1 JCK-1
......................uggaucuuguuGgacagcacu............................................................................................................................................................................................................ 10 1 JCK-1
......................uUgaucuuguuugacagcacu............................................................................................................................................................................................................ 2 1 LCK-3
......................uAgaucuuguuugacagcacu............................................................................................................................................................................................................ 22 1 LT-3
......................ugCaucuuguuugacagcacu............................................................................................................................................................................................................ 2 1 LT-2
......................uggaucuuguuugaAagcacu............................................................................................................................................................................................................ 2 1 JCK-1
......................uggaucuuguuugacagAacu............................................................................................................................................................................................................ 3 1 LT-1
......................uggaucuuguuugacagcaAu............................................................................................................................................................................................................ 4 1 LT-2
......................uggaucCuguuugacagcacu............................................................................................................................................................................................................ 12 1 JCK-2
......................uggaucuuguuugacagcacG............................................................................................................................................................................................................ 12 1 LT-3
......................uggauAuuguuugacagcacu............................................................................................................................................................................................................ 2 1 JCK-2
......................uUgaucuuguuugacagcacu............................................................................................................................................................................................................ 7 1 LT-2
......................uggaucuuguuugacagcCcu............................................................................................................................................................................................................ 29 1 LT-1
......................uggaucuuguuugaAagcacu............................................................................................................................................................................................................ 4 1 JCK-2
......................uNgaucuuguuugacagcacu............................................................................................................................................................................................................ 4 1 JT-1
......................uggaucuuguCugacagcacu............................................................................................................................................................................................................ 7 1 JCK-3
......................uggCucuuguuugacagcacu............................................................................................................................................................................................................ 92 1 JCK-1
......................uggaucuuguuugacagcCcu............................................................................................................................................................................................................ 4 1 LCK-2
......................uggaucuuguuugacagcacu............................................................................................................................................................................................................ 20263 0 LCK-3
......................uggaCcuuguuugacagcacu............................................................................................................................................................................................................ 4 1 LT-3
......................uggaucuuguuugCcagcacu............................................................................................................................................................................................................ 36 1 LCK-3
......................uggaucuuguuugacCgcacu............................................................................................................................................................................................................ 12 1 LT-1
......................uggaucuuguuugacaCcacu............................................................................................................................................................................................................ 5 1 JCK-1
......................uCgaucuuguuugacagcacu............................................................................................................................................................................................................ 1 1 LCK-3
......................uggaucuuguuugacagcCcu............................................................................................................................................................................................................ 17 1 JCK-3
......................uggCucuuguuugacagcacu............................................................................................................................................................................................................ 118 1 JCK-3
......................uggaucuuguuugacagcacN............................................................................................................................................................................................................ 22 1 JCK-1
......................uggaCcuuguuugacagcacu............................................................................................................................................................................................................ 1 1 LCK-3
```

```
                      Mature                                                                                 Star
uccaucuuguucguuuacccucuggaucuuguuugacagcacucucuuauauuuugccauucaucuuuauaugccaaauggcgaugaugaugauggugggagugcuaucgacaugauccagaaggugcagucgaauaagauacagagggaccacccacaguaccuacucucguuacaaucguauucuucuccucacuuauuauuauuauuauuauuauuccgaucauuucuuuuaaucuauugaag
......................uggaCcuuguuugacagcacu...........................................................................................................................................................................................   2     1   LT-2
......................uggaucuuguuugacagAacu...........................................................................................................................................................................................   1     1   JCK-2
......................uggaucuuguuugacCgcacu...........................................................................................................................................................................................   7     1   LCK-3
......................uggaucuuguuugacagGacu...........................................................................................................................................................................................   24    1   JCK-1
......................uggaucuuguuugacagcCcu...........................................................................................................................................................................................   31    1   LT-3
......................uggaucuuguuugacCgcacu...........................................................................................................................................................................................   29    1   JCK-2
......................uggaucuuguuugNcagcacu...........................................................................................................................................................................................   15    1   LCK-1
......................uNgaucuuguuugacagcacu...........................................................................................................................................................................................   3     1   LT-1
......................ugAaucuuguuugacagcacu...........................................................................................................................................................................................   3     1   LCK-2
......................uggaucuuguuugGcagcacu...........................................................................................................................................................................................   5     1   LCK-1
......................uNgaucuuguuugacagcacu...........................................................................................................................................................................................   55    1   JCK-3
......................uggaucuCguuugacagcacu...........................................................................................................................................................................................   3     1   LCK-3
......................uggaucuCguuugacagcacu...........................................................................................................................................................................................   5     1   JCK-1
......................uCgaucuuguuugacagcacu...........................................................................................................................................................................................   1     1   JCK-3
......................uggaucuuguuugCcagcacu...........................................................................................................................................................................................   67    1   LT-3
......................uggauGuuguuugacagcacu...........................................................................................................................................................................................   1     1   JCK-3
......................uggaucuuguuAgacagcacu...........................................................................................................................................................................................   1     1   LCK-3
......................uggaucuuguuugacagUacu...........................................................................................................................................................................................   34    1   JCK-2
......................uggCucuuguuugacagcacu...........................................................................................................................................................................................   2     1   JT-2
......................uggaucuuguuugacagGacu...........................................................................................................................................................................................   2     1   JCK-3
......................uggaucGuguuugacagcacu...........................................................................................................................................................................................   2     1   JCK-1
......................uggaucuuguuugacCgcacu...........................................................................................................................................................................................   3     1   JT-1
......................uggaucuuguuugacCgcacu...........................................................................................................................................................................................   26    1   LT-2
......................uAgaucuuguuugacagcacu...........................................................................................................................................................................................   14    1   LT-1
......................uggaucuuguuugaGagcacu...........................................................................................................................................................................................   8     1   LCK-1
......................uggaucuuguuugacagcaAu...........................................................................................................................................................................................   1     1   LT-1
......................uggaucuuguuugacagcNcu...........................................................................................................................................................................................   22    1   JCK-1
......................uggGucuuguuugacagcacu...........................................................................................................................................................................................   8     1   JCK-2
......................uggaucuuguuugacGgcacu...........................................................................................................................................................................................   2     1   LCK-3
......................uggaucuugGuugacagcacu...........................................................................................................................................................................................   1     1   JCK-2
......................uggaucuuguuugacagUacu...........................................................................................................................................................................................   11    1   LT-2
......................uggaucuuguuugacagcaUu...........................................................................................................................................................................................   1732  1   JCK-3
......................uggaucuugCuugacagcacu...........................................................................................................................................................................................   2     1   LT-1
......................ugAaucuuguuugacagcacu...........................................................................................................................................................................................   8     1   JCK-3
......................uggaucuuguuugCcagcacu...........................................................................................................................................................................................   86    1   JCK-1
......................uggauUuuguuugacagcacu...........................................................................................................................................................................................   1     1   LT-2
......................uggaucuuguuugacaCcacu...........................................................................................................................................................................................   5     1   JCK-3
......................uggauUuuguuugacagcacu...........................................................................................................................................................................................   4     1   JCK-3
......................uggaucuuguCugacagcacu...........................................................................................................................................................................................   1     1   LT-2
......................uAgaucuuguuugacagcacu...........................................................................................................................................................................................   5     1   JCK-1
......................uNgaucuuguuugacagcacu...........................................................................................................................................................................................   104   1   JCK-2
......................uggaucuuguuGgacagcacu...........................................................................................................................................................................................   1     1   JCK-3
......................uggaucuuguuugacagcUcu...........................................................................................................................................................................................   6     1   LT-2
......................uggauUuuguuugacagcacu...........................................................................................................................................................................................   2     1   LT-3
......................uggaucuuguuugacagcaAu...........................................................................................................................................................................................   3     1   LT-3
......................uggaucuuguuugGcagcacu...........................................................................................................................................................................................   3     1   JCK-2
......................uggaucuuguuuUacagcacu...........................................................................................................................................................................................   3     1   LT-3
......................uggaCcuuguuugacagcacu...........................................................................................................................................................................................   1     1   LCK-2
......................uggaGcuuguuugacagcacu...........................................................................................................................................................................................   2     1   JCK-3
......................uggaucuuAuuugacagcacu...........................................................................................................................................................................................   3     1   LT-3
......................uggaucuuUuuugacagcacu...........................................................................................................................................................................................   4     1   JCK-2
......................uggaucuuUuuugacagcacu...........................................................................................................................................................................................   6     1   JCK-3
......................uggaucuuguuuUacagcacu...........................................................................................................................................................................................   4     1   LT-1
......................Cggaucuuguuugacagcacu...........................................................................................................................................................................................   1     1   JT-1
......................uggaucuuguuugacagcacu...........................................................................................................................................................................................   1709  0   JT-1
......................uggaCcuuguuugacagcacu...........................................................................................................................................................................................   13    1   JCK-3
......................uggaucuugGuugacagcacu...........................................................................................................................................................................................   1     1   JCK-1
......................uggaucuuguuuAacagcacu...........................................................................................................................................................................................   3     1   LT-1
......................uggCucuuguuugacagcacu...........................................................................................................................................................................................   152   1   JCK-2
......................uggaucuuguuugacagcUcu...........................................................................................................................................................................................   1     1   JCK-2
......................uggaucuuguuuAacagcacu...........................................................................................................................................................................................   6     1   JCK-1
......................uggaucuuguuugacagcaUu...........................................................................................................................................................................................   20    1   LT-1
......................uggaucuuguuugacagcGcu...........................................................................................................................................................................................   2     1   JCK-1
......................ugUaucuuguuugacagcacu...........................................................................................................................................................................................   4     1   JCK-3
......................ugCaucuuguuugacagcacu...........................................................................................................................................................................................   1     1   LCK-3
......................uggaucuuguuugacagUacu...........................................................................................................................................................................................   5     1   LT-3
......................uggCucuuguuugacagcacu...........................................................................................................................................................................................   9     1   LCK-2
......................uNgaucuuguuugacagcacu...........................................................................................................................................................................................   66    1   JCK-1
......................uggaucuuguuugacGgcacu...........................................................................................................................................................................................   2     1   LT-1
......................uggaucuuguuuNacagcacu...........................................................................................................................................................................................   1     1   LCK-1
```

Mature / Star			
uccaucuuguucguuuacccucuggaucuuguuugacagcacucucuuauauuuugccauucaucuuuauaugccaaauggcgaugaugaugaugguggggagugcuaucgacaugauccagaaggugcagucgaauaagauacagagggaccacccacaguaccuacucucguuacaaucguauucuucuccucacuuauuauuauuauuauuauuauuccgaucauuucuuuuaaucuauugaag			
......................uggNucuuguuugacagcacu...	1	1	LCK-1
......................uggaucuuguuugCcagcacu...	7	1	LCK-2
......................uggaucuugCuugacagcacu...	1	1	LT-3
......................uggaucuuguuugacagcCcu...	34	1	LT-2
......................uggaucuuguuAgacagcacu...	1	1	JCK-1
......................uggaucuuguuugacCgcacu...	19	1	JCK-1
......................uggaucuuguCugacagcacu...	6	1	JCK-1
......................uggaucuuguuugCcagcacu...	5	1	JT-1
......................uggaucuCguuugacagcacu...	1	1	LT-1
......................uggaucuuguuugacagcacu...	27651	0	LT-3
......................Cggaucuuguuugacagcacu...	6	1	JCK-1
......................uggaucuuguuugacGgcacu...	7	1	LT-2
......................uggaucuuguuugacagUacu...	1	1	JT-2
......................Cggaucuuguuugacagcacu...	7	1	JCK-3
......................uggGucuuguuugacagcacu...	5	1	JCK-3
......................uggaucuuguuAgacagcacu...	1	1	JCK-2
......................uggaucuugCuugacagcacu...	6	1	JCK-3
......................uggaucuuCuuugacagcacu...	1	1	JCK-2
......................uggaucuuguGugacagcacu...	15	1	JCK-1
......................uggaucuuguuugacagcacu...	23834	0	LT-1
......................uggaucuuguuugacGgcacu...	1	1	JT-3
......................uggaucuuguuugacagcacG...	10	1	JCK-1
......................uggaucuuAuuugacagcacu...	4	1	JCK-3
......................ugAaucuuguuugacagcacu...	19	1	JCK-2
......................uggGucuuguuugacagcacu...	1	1	JT-1
......................Cggaucuuguuugacagcacu...	1	1	JT-3
......................ugAaucuuguuugacagcacu...	3	1	JCK-1
......................uggaucuuUuuugacagcacu...	1	1	LT-3
......................uggaucuuguuugaAagcacu...	5	1	JCK-3
......................uggGucuuguuugacagcacu...	8	1	JCK-1
......................uggaucuuguuugacagcaUu...	68	1	LT-2
......................uggaucuuguuuAacagcacu...	1	1	LT-3
......................uUgaucuuguuugacagcacu...	4	1	JCK-2
......................uAgaucuuguuugacagcacu...	13	1	LCK-3
......................ugCaucuuguuugacagcacu...	1	1	JCK-3
......................uggCucuuguuugacagcacu...	65	1	LT-1
......................uggaucuugGuugacagcacu...	1	1	JCK-3
......................uggaucuuguCugacagcacu...	5	1	JT-2
......................uggaucuuguuugCcagcacu...	105	1	JCK-3
......................uggaucuuguuugGcagcacu...	1	1	LT-3
......................uggaucuuguuugCcagcacu...	5	1	LCK-1
......................uggaucuuguuugacGgcacu...	7	1	JCK-1
......................uggaucuuguuugacagcacG...	1	1	JT-2
......................uggaucuuguuugacagcaUu...	64	1	LT-3
......................ugAaucuuguuugacagcacu...	11	1	LT-3
......................uggaucuuguuugacagcCcu...	4	1	LCK-1
......................uggaucCuguuugacagcacu...	1	1	LCK-1
......................uggaucuuguuuUacagcacu...	6	1	JCK-1
......................uggaucuuguuugacagcaGu...	2	1	JCK-1
......................ugAaucuuguuugacagcacu...	2	1	LCK-1
......................uggaucCuguuugacagcacu...	2	1	LT-2
......................uggaucuuguuugacCgcacu...	3	1	LCK-1
......................uggaucuuguuugacagcaAu...	1	1	LCK-2
......................uggaucuuCuuugacagcacu...	1	1	JCK-3
......................uggaucuuguuuAacagcacu...	7	1	JCK-3
......................uggaucuuguuuCacagcacu...	2	1	JCK-1
......................uggaucuuguuugacagcaUu...	55	1	JT-1
......................uggaucuuguuugacagcacG...	13	1	JCK-3
......................uggNucuuguuugacagcacu...	1	1	JCK-2
......................uggCucuuguuugacagcacu...	8	1	LCK-1
......................uggaucuuguuuAacagcacu...	2	1	LT-2
......................uAgaucuuguuugacagcacu...	15	1	JCK-3
......................uggaucuuguuugacagcaUu...	1426	1	JCK-1
......................uggaucCuguuugacagcacu...	10	1	JCK-1
......................uggaucuuguuugacagcacu...	66730	0	JCK-2
......................uggaucuuAuuugacagcacu...	9	1	LT-2
......................uggaCcuuguuugacagcacu...	3	1	LT-1
......................uggCucuuguuugacagcacu...	7	1	JT-1
......................uggaucuuguCugacagcacu...	1	1	LCK-2
......................uggGucuuguuugacagcacu...	3	1	LT-3

```
                      Mature                                                                              Star
uccaucuuguucguuuacccucuggaucuuguuugacagcacucucuuauauuuugccauucaucuuuauaugccaaauggcgaugaugaugaugguggagugcuaucgacaugauccagaaggugcagucgaauaagauacagagggaccacccacaguaccuacucucguuacaaucguauucuucuccucacuuauuauuauuauuauuauuauuccgaucauuuucuuuuaaucuauugaag
......................uggaucuuguuugacagcaAu.............................................................................................................................................................................................................. 5     1     JT-2
......................uggaucuuUuuugacagcacu.............................................................................................................................................................................................................. 2     1     LT-1
......................uAgaucuuguuugacagcacu.............................................................................................................................................................................................................. 30    1     LT-2
......................uggaucuuguuuAacagcacuc............................................................................................................................................................................................................. 1     1     JCK-1
......................uggaucuuguuugacagcacuA............................................................................................................................................................................................................. 33    1     LT-2
......................uggaucuuguuugacagcCcuc............................................................................................................................................................................................................. 9     1     JCK-3
......................uggaucuuguuugacagcacuU............................................................................................................................................................................................................. 48    1     LT-3
......................uggGucuuguuugacagcacuc............................................................................................................................................................................................................. 1     1     JCK-3
......................uggaucuuguuugacagcacuG............................................................................................................................................................................................................. 11    1     JCK-1
......................uggGucuuguuugacagcacuc............................................................................................................................................................................................................. 1     1     JCK-1
......................uggCucuuguuugacagcacuc............................................................................................................................................................................................................. 3     1     LCK-3
......................uggaucuugCuugacagcacuc............................................................................................................................................................................................................. 1     1     JCK-2
......................uggaucuuguuugCcagcacuc............................................................................................................................................................................................................. 7     1     LT-1
......................uggaucuuguuugacagcacuU............................................................................................................................................................................................................. 85    1     JCK-3
......................uggaucuuguuugCcagcacuc............................................................................................................................................................................................................. 2     1     LT-3
......................uggaucuuAuuugacagcacuc............................................................................................................................................................................................................. 4     1     JCK-2
......................uggaucuuguuugacagcUcuc............................................................................................................................................................................................................. 2     1     JCK-2
......................uggCucuuguuugacagcacuc............................................................................................................................................................................................................. 1     1     JT-2
......................uggaucuuguuugacagcacuc............................................................................................................................................................................................................. 152   0     JT-3
......................uggaucuuguuugacagcacuc............................................................................................................................................................................................................. 70    0     LCK-2
......................uggaucuuguuugacagcacuG............................................................................................................................................................................................................. 3     1     LT-3
......................uggaucuuguuugaAagcacuc............................................................................................................................................................................................................. 4     1     JCK-2
......................uggGucuuguuugacagcacuc............................................................................................................................................................................................................. 4     1     JCK-2
......................ugAaucuuguuugacagcacuc............................................................................................................................................................................................................. 2     1     LT-3
......................uggaucuuguuugacCgcacuc............................................................................................................................................................................................................. 13    1     JCK-2
......................uggaucCuguuugacagcacuc............................................................................................................................................................................................................. 2     1     JCK-2
......................uggaucuuguuugacagcacuU............................................................................................................................................................................................................. 97    1     JCK-2
......................uggaGcuuguuugacagcacuc............................................................................................................................................................................................................. 1     1     JCK-3
......................uggaucuuguuugCcagcacuc............................................................................................................................................................................................................. 44    1     JCK-2
......................uggaucuuguuuUacagcacuc............................................................................................................................................................................................................. 1     1     JCK-1
......................uggaucuuguuugacagcaUuc............................................................................................................................................................................................................. 7     1     JCK-1
......................ugUaucuuguuugacagcacuc............................................................................................................................................................................................................. 1     1     JCK-1
......................uggauUuuguuugacagcacuc............................................................................................................................................................................................................. 7     1     JCK-2
......................uggaucuuguuugacagcacuG............................................................................................................................................................................................................. 1     1     LT-2
......................uUgaucuuguuugacagcacuc............................................................................................................................................................................................................. 1     1     JCK-3
......................uggaucuuguuugacagcaNuc............................................................................................................................................................................................................. 11    1     JCK-1
......................uggCucuuguuugacagcacuc............................................................................................................................................................................................................. 2     1     LCK-1
......................Cggaucuuguuugacagcacuc............................................................................................................................................................................................................. 1     1     LT-1
......................uggaucuuguuugaGagcacuc............................................................................................................................................................................................................. 4     1     JCK-2
......................uggaucuuguuugacagGacuc............................................................................................................................................................................................................. 7     1     JCK-1
......................ugNaucuuguuugacagcacuc............................................................................................................................................................................................................. 2     1     LT-2
......................uggaucuuguuuAacagcacuc............................................................................................................................................................................................................. 1     1     JCK-3
......................uggaucuuguuugacagcaUuc............................................................................................................................................................................................................. 8     1     JCK-3
......................uggaucuuguuugCcagcacuc............................................................................................................................................................................................................. 3     1     LCK-3
......................uggaucuuguuugacagcacuc............................................................................................................................................................................................................. 138   0     JT-2
......................uggaucuuguuugacagcacuG............................................................................................................................................................................................................. 7     1     LT-1
......................uggaucuuguuugacaAcacuc............................................................................................................................................................................................................. 6     1     JCK-3
......................uggCucuuguuugacagcacuc............................................................................................................................................................................................................. 7     1     LT-3
......................ugCaucuuguuugacagcacuc............................................................................................................................................................................................................. 1     1     JCK-2
......................uggaucuCguuugacagcacuc............................................................................................................................................................................................................. 2     1     JCK-1
......................uggaucuuguuugacagcacuc............................................................................................................................................................................................................. 627   0     LT-2
......................uggaucuuguuugacagcacuc............................................................................................................................................................................................................. 1262  0     LT-1
......................uggaucuuguuugacagcacGc............................................................................................................................................................................................................. 1     1     JCK-3
......................uggaucuuguGugacagcacuc............................................................................................................................................................................................................. 5     1     JCK-1
......................uggaucuuguuugacagcacuG............................................................................................................................................................................................................. 4     1     JCK-2
......................uggaucuuguuugacagcacuc............................................................................................................................................................................................................. 1069  0     LT-3
......................uggaucuuguuGgacagcacuc............................................................................................................................................................................................................. 3     1     JCK-1
......................uggaucuuguuugacagcacuU............................................................................................................................................................................................................. 22    1     LCK-2
......................uggaucuuguuugacagcacuc............................................................................................................................................................................................................. 9941  0     JCK-1
......................uggaucuuguuugacagcacuA............................................................................................................................................................................................................. 38    1     JCK-1
......................uggaucuuguuugacagcacuA............................................................................................................................................................................................................. 12    1     LCK-3
......................uggaucuuguuuUacagcacuc............................................................................................................................................................................................................. 1     1     JCK-3
......................uggCucuuguuugacagcacuc............................................................................................................................................................................................................. 36    1     JCK-2
......................ugUaucuuguuugacagcacuc............................................................................................................................................................................................................. 1     1     JCK-2
......................uggaucuuguuugacagcacGc............................................................................................................................................................................................................. 1     1     JCK-2
......................uggaucuuguuugacagcacuA............................................................................................................................................................................................................. 32    1     LT-1
......................uggaucuuguuugacagcacuU............................................................................................................................................................................................................. 64    1     LCK-3
......................uggaucuuguuugacagcacuA............................................................................................................................................................................................................. 41    1     JCK-3
......................uggaucuuguuugacagcacuA............................................................................................................................................................................................................. 34    1     LT-3
......................uggaucuuguuugacCgcacuc............................................................................................................................................................................................................. 5     1     JCK-1
```

```
                      Mature                                                                                   Star
uccaucuuguucguuuacccucuggaucuuguuugacagcacucucuuauauuuugccauucaucuuuauaugccaaauggcgaugaugaugauggugggagugcuaucgacaugauccagaaggugcagucgaauaagauacagagggaccacccacaguaccuacucucguuacaaucguauucuucuccucacuuauuauuauuauuauuauuauuauuccgaucauuucuuuuaaucuauugaag
......................uggaucuuguuugacaAcacuc..............................................................................................................................................................................................................   4      1   JCK-1
......................uggaucuuguuugCcagcacuc..............................................................................................................................................................................................................   2      1   JT-1
......................uggauUuuguuugacagcacuc..............................................................................................................................................................................................................   1      1   JCK-3
......................uggaucuuguuugacagNacuc..............................................................................................................................................................................................................   1      1   JCK-3
......................uggaucuuguuugacagcGcuc..............................................................................................................................................................................................................   1      1   JCK-1
......................uNgaucuuguuugacagcacuc..............................................................................................................................................................................................................   15     1   JCK-3
......................uAgaucuuguuugacagcacuc..............................................................................................................................................................................................................   7      1   JCK-2
......................ugUaucuuguuugacagcacuc..............................................................................................................................................................................................................   3      1   JCK-3
......................uggaucuuguuugacagcacGc..............................................................................................................................................................................................................   1      1   JCK-1
......................uggaucuugGuugacagcacuc..............................................................................................................................................................................................................   3      1   JCK-3
......................uggaucuuguuugacagcaAuc..............................................................................................................................................................................................................   1      1   JCK-2
......................uggaucuuguuugacagcacuc..............................................................................................................................................................................................................   12872  0   JCK-3
......................uggaucuuguuugacagcCcuc..............................................................................................................................................................................................................   1      1   LCK-1
......................Cggaucuuguuugacagcacuc..............................................................................................................................................................................................................   1      1   LT-2
......................uggaucuuUuuugacagcacuc..............................................................................................................................................................................................................   2      1   JCK-2
......................Cggaucuuguuugacagcacuc..............................................................................................................................................................................................................   6      1   JCK-2
......................uggaucuuguuugacagcGcuc..............................................................................................................................................................................................................   3      1   JCK-2
......................uggCucuuguuugacagcacuc..............................................................................................................................................................................................................   33     1   JCK-3
......................uggaucuuguuugacCgcacuc..............................................................................................................................................................................................................   3      1   JCK-3
......................uggaucuuguuugacagcacuc..............................................................................................................................................................................................................   76     0   LCK-1
......................uggNucuuguuugacagcacuc..............................................................................................................................................................................................................   7      1   JCK-1
......................uggaucuuguuugNcagcacuc..............................................................................................................................................................................................................   1      1   LCK-1
......................uggaucuuguuugCcagcacuc..............................................................................................................................................................................................................   19     1   JCK-3
......................uggaucuuguuugacagcacuU..............................................................................................................................................................................................................   47     1   LT-1
......................uggaucuuAuuugacagcacuc..............................................................................................................................................................................................................   1      1   LT-2
......................uggaucuuguuugacagcaUuc..............................................................................................................................................................................................................   5      1   JCK-2
......................uggaucuuguuugacaAcacuc..............................................................................................................................................................................................................   3      1   JCK-2
......................uggaucuuguuugacagcacuU..............................................................................................................................................................................................................   68     1   JCK-1
......................uggCucuuguuugacagcacuc..............................................................................................................................................................................................................   1      1   JT-1
......................uggaucuuguuugacagcacuU..............................................................................................................................................................................................................   27     1   LT-2
......................Cggaucuuguuugacagcacuc..............................................................................................................................................................................................................   1      1   LCK-3
......................uggaucuuguuuAacagcacuc..............................................................................................................................................................................................................   3      1   JCK-2
......................uggaCcuuguuugacagcacuc..............................................................................................................................................................................................................   6      1   JCK-1
......................uggaucuuguuugacagcNcuc..............................................................................................................................................................................................................   7      1   JCK-1
......................Cggaucuuguuugacagcacuc..............................................................................................................................................................................................................   5      1   JCK-3
......................uggaucuuguuugacagcacuN..............................................................................................................................................................................................................   4      1   LT-2
......................uAgaucuuguuugacagcacuc..............................................................................................................................................................................................................   2      1   LT-1
......................uUgaucuuguuugacagcacuc..............................................................................................................................................................................................................   1      1   JCK-2
......................ugAaucuuguuugacagcacuc..............................................................................................................................................................................................................   2      1   JCK-1
......................uNgaucuuguuugacagcacuc..............................................................................................................................................................................................................   17     1   JCK-2
......................uggaucuuguuugacGgcacuc..............................................................................................................................................................................................................   1      1   LT-1
......................ugAaucuuguuugacagcacuc..............................................................................................................................................................................................................   1      1   LT-2
......................uggaucuuUuuugacagcacuc..............................................................................................................................................................................................................   1      1   JCK-3
......................uggaucuuAuuugacagcacuc..............................................................................................................................................................................................................   4      1   JCK-3
......................uggaCcuuguuugacagcacuc..............................................................................................................................................................................................................   9      1   JCK-3
......................uggaucuuguuugacagcCcuc..............................................................................................................................................................................................................   1      1   JT-3
......................uggaucuuguuugGcagcacuc..............................................................................................................................................................................................................   3      1   JCK-2
......................uNgaucuuguuugacagcacuc..............................................................................................................................................................................................................   26     1   JCK-1
......................uggaucuugCuugacagcacuc..............................................................................................................................................................................................................   1      1   JCK-3
......................uggaucuuguuNgacagcacuc..............................................................................................................................................................................................................   29     1   JCK-1
......................uggaucuuguuugacagcaUuc..............................................................................................................................................................................................................   3      1   LT-2
......................ugAaucuuguuugacagcacuc..............................................................................................................................................................................................................   4      1   JCK-2
......................uggaCcuuguuugacagcacuc..............................................................................................................................................................................................................   1      1   LT-2
......................uggaucuuguuugacagcacuc..............................................................................................................................................................................................................   520    0   LCK-3
......................uggaucuuguuugacagcacuA..............................................................................................................................................................................................................   59     1   JCK-2
......................uggaucuuguuugacCgcacuc..............................................................................................................................................................................................................   2      1   LCK-3
......................uggaucuuguuugacagcacNc..............................................................................................................................................................................................................   3      1   JCK-1
......................uAgaucuuguuugacagcacuc..............................................................................................................................................................................................................   10     1   JCK-3
......................uUgaucuuguuugacagcacuc..............................................................................................................................................................................................................   1      1   JCK-1
......................uggaucuuguuugacagUacuc..............................................................................................................................................................................................................   5      1   JCK-2
......................uggaucuuguuugacagcCcuc..............................................................................................................................................................................................................   7      1   JCK-2
......................uggaucuugGuugacagcacuc..............................................................................................................................................................................................................   3      1   JCK-1
......................Cggaucuuguuugacagcacuc..............................................................................................................................................................................................................   2      1   JCK-1
......................uggaucuuguuugacagcacuU..............................................................................................................................................................................................................   18     1   LCK-1
......................uggaucuCguuugacagcacuc..............................................................................................................................................................................................................   3      1   JCK-2
......................uggaucuuguuugGcagcacuc..............................................................................................................................................................................................................   1      1   JCK-1
......................uggaucuuguuugacagcacuG..............................................................................................................................................................................................................   2      1   JCK-3
......................uAgaucuuguuugacagcacuc..............................................................................................................................................................................................................   1      1   LT-2
......................uggaucuugGuugacagcacuc..............................................................................................................................................................................................................   3      1   JCK-2
......................uggaucuuguuugacCgcacuc..............................................................................................................................................................................................................   1      1   LT-1
```

Mature **Star**

uccaucuuguucguuuacccucuggaucuuguuugacagcacucucuuauauuuugccauucaucuuuauaugccaaauggcgaugaugaugauggugggagugcuaucgacaugauccagaaggugcagucgaauaagauacagagggaccacccacaguaccuacucucguuacaaucguauucuucuccucacuuauuauuauuauuauuauuauuauuccgaucauuuucuuuuaaucuauugaag

......................uggaucuuguuugCcagcacuc...	24	1	JCK-1
......................uggCucuuguuugacagcacuc...	4	1	LT-1
......................uggaucCuguuugacagcacuc...	1	1	JCK-1
......................uggaucuuguuugacagcacuc...	17179	0	JCK-2
......................uAgaucuuguuugacagcacuc...	1	1	LT-3
......................uggCucuuguuugacagcacuc...	36	1	JCK-1
......................uggaucuuguuugacagcCcuc...	7	1	JCK-1
......................uggaucCuguuugacagcacuc...	5	1	JCK-3
......................uggaucuCguuugacagcacuc...	2	1	JCK-3
......................uAgaucuuguuugacagcacuc...	1	1	LCK-3
......................uggaucuuguuugCcagcacuc...	1	1	JT-2
......................uggaucuuguuugacCgcacuc...	1	1	LT-2
......................uggaucuuguuugacGgcacuc...	2	1	JCK-3
......................uggCucuuguuugacagcacuc...	4	1	LT-2
......................uggaucuuNuuugacagcacuc...	1	1	JCK-3
......................uggauUuuguuugacagcacuc...	5	1	JCK-1
......................uggaucuuguuugCcagcacuc...	2	1	LT-2
......................uggaucuuguuugacagcacuU...	1	1	JT-2
......................uAgaucuuguuugacagcacuc...	3	1	JCK-1
......................uggaucuuguuugacagcacuU...	4	1	JT-3
......................uggaucuuguCugacagcacuc...	1	1	JCK-1
......................uggaucuuguuugacagcacuc...	220	0	JT-1
......................uUgaucuuguuugacagcacuc...	1	1	LT-2
......................uggaucuuguuugacagUacuc...	1	1	JCK-1
......................ugAaucuuguuugacagcacuc...	1	1	LT-1
......................uggauAuuguuugacagcacuc...	1	1	JCK-1
......................ugAaucuuguuugacagcacuc...	2	1	JCK-3
......................uggaucuuguuugacaCcacuc...	1	1	JCK-3
......................uggaucuuguuugacagcUcuc...	1	1	JCK-1
......................uggaucuuguuugacagcacuN...	9	1	JCK-1
......................uggaucuuguuugacagcacuA...	11	1	JT-1
......................uggaucuuguuugacagcacucG...	3	1	JCK-1
......................uggaucuuguuugacagcacucG...	4	1	JCK-3
......................uggaucuuguuugacagcacuGu...	1	1	LT-3
......................uggaucuuguuugacagcacucA...	10	1	JT-2
......................uggaucuuguuugacagcacuGu...	2	1	LT-2
......................uggaucuuguuugacagcacucu...	90	0	JCK-1
......................uggaucuuguuugacagcacucu...	111	0	JCK-2
......................uggaucuuguuugacagcacucu...	14	0	LT-2
......................uggaucuuguuugacagcacucA...	14	1	LT-2
......................uggaucuuguuugacagcacucA...	6	1	LT-3
......................uggaucuuguuugacagcacucA...	79	1	JCK-1
......................uggaucuuguuugacagcacuUu...	23	1	LCK-1
......................uggaucuuguuugacagcacuGu...	5	1	LT-1
......................uggaucuuguuugacagcacuGu...	1	1	JCK-1
......................uggaucuuguuugacagcacucu...	26	0	LT-1
......................uggaucuuguuugacagcacuUu...	62	1	LT-1
......................uggaucuuguuugacagcacuUu...	159	1	JCK-2
......................uggaucuuguuugacagcacucu...	16	0	LCK-3
......................uggaucuuguuugacagcacucu...	80	0	JCK-3
......................uggaucuuguuNgacagcacucu...	3	1	JCK-1
......................uggaucuuguuugacagcacuUu...	39	1	JT-1
......................uggaucuuguuugacagcacuAu...	1	1	JCK-3
......................uggaucuuguuugacagcacucA...	24	1	LCK-3
......................uggaucuuguuugacagcacucA...	140	1	JCK-2
......................uggCucuuguuugacagcacucu...	1	1	LT-2
......................uggaucuuguuugacagcacuUu...	53	1	LT-3
......................uggaucuuguuugacagcacuAu...	2	1	JCK-2
......................uggaucuuguuugacagcacuUu...	71	1	LT-2
......................uggaucuuguuugacagcacuUu...	21	1	JT-2
......................uggaucuuguuugacagcacuUu...	21	1	JT-3
......................uggaucuuguuugacagcacuGu...	6	1	JCK-2
......................uggaucuuguuugacagcacuUu...	19	1	LCK-2
......................uggaucuuguuugacagcacuUu...	75	1	LCK-3
......................uggaucuuguuugacagcacuUu...	109	1	JCK-1
......................uggaucuuguuugacagcacuUu...	112	1	JCK-3
......................uggaucuuguuugacagcacuAu...	3	1	JCK-1
......................uggaucuuguuugacagcacucu...	10	0	LT-3
......................uggaucuuguuugacagcacucG...	5	1	JCK-2
......................uggaucuuguuugacagcacucA...	100	1	JCK-3

Mature Star

uccaucuuguucguuuacccucuggaucuuguuugacagcacucucuuauauuuugccauucaucuuuauaugccaaauggcgaugaugaugauggugggagugcuaucgacaugauccagaaggugcagucgaauaagauacagagggaccacccacaguaccuacucucguuacaaucguauucuucuccucacuuauuauuauuauuauuauuauuauuauuccgaucauuuucuuuuaaucuauugaag			
......................uggaucuuguuugacagcacucA....	14	1	JT-3
......................uggaucuuguuugacagcacucA....	23	1	LT-1
......................uggaucuuguuugacagcacucuU....	1	1	JCK-1
......................uggaucuuguuugacagcacucuc....	12	0	JCK-1
......................uggaucuuguuugacagcacucuc....	19	0	JCK-3
......................uggaucuuguuugacagcacucuc....	1	0	LT-1
......................uggaucuuguuugacagcacucuA....	1	1	LT-1
......................uggaucuuguuugacagcacucuU....	7	1	LT-2
......................uggaucuuguuugacagcacucuU....	6	1	JCK-2
......................uggaucuuguuugacagcacucuU....	9	1	LT-1
......................uggaucuuguuugacagcacucuc....	3	0	LCK-3
......................uggaucuuguuugacagcacucuc....	19	0	JCK-2
......................uggaucuuguuugacagcacucuA....	1	1	LT-3
......................uggaucuuguuugacagcacuGuc....	1	1	LT-3
......................uggaucuuguuugacagcacucuA....	2	1	JCK-3
......................uggaucuuguuugacagcacucuA....	2	1	JCK-2
......................uggaucuuguuugacagcacucuc....	1	0	LT-3
......................uggaucuuguuugacagcacucuA....	5	1	LT-2
......................uggaucuuguuNgacagcacucuc....	1	1	JCK-1
......................uggaucuuguuugacagcacucuA....	2	1	JCK-1
......................uggaucuuguuugacagcacucAc....	13	1	LCK-3
......................uggaucuuguuugacagcacucAc....	2	1	LT-3
......................uggaucuuguuugacagcacucAc....	7	1	LT-1
......................uggaucuuguuugacagcacuUuc....	3	1	JCK-2
......................uggaucuuguuugacagcacucucu....	2	0	JCK-3
......................uggaucuuguuugacagcacucucu....	6	0	JCK-2
......................uggaucuuguuugacagcacucuUu....	6	1	LT-2
......................uggaucuuguuugacagcacucAcu....	1	1	JCK-2
......................uggaucuuguuugacagcacucuUu....	2	1	JCK-2
......................uggaucuuguuugacagcacucuUu....	1	1	JCK-1
......................uggaucuuguuugacagcacucuAu....	1	1	LT-1
......................uggaucuuguuugacagcacucucu....	1	0	JCK-1
......................uggaucuuguuugacagcacucucu....	1	0	LT-3
......................uggaucuuguuugacagcacucuUu....	1	1	LT-1
......................uggaucuuguuugacagcacucuAuu....	1	1	JCK-1
.......................ggaucuuguuugacagca....	1	0	LT-1
.......................ggaucuuguuugacagca....	2	0	JCK-3
.......................ggaucuuguuugacagca....	1	0	JCK-1
.......................ggaucuuguuugacagcac....	1	0	LT-2
.......................ggaucuuguuugacagcac....	2	0	LT-1
.......................ggaucuuguuugacagcac....	4	0	LCK-3
.......................ggaucuuguuugacagcacu....	4	0	JCK-3
.......................ggaucuuguuugacagcacu....	2	0	LT-3
.......................ggaucuuguuugacagcacu....	2	0	LCK-3
.......................ggaucuuguuugacagcacu....	3	0	JCK-2
.......................ggaucuuguuugacagcacu....	5	0	LT-1
.......................ggaucuuguuugacagcacu....	5	0	JCK-1
.......................ggaucuuguuugacagcacu....	5	0	LT-2
.......................ggaucuuguuugacagcacuc....	6	0	JCK-2
.......................ggaucuuguuugacagcacuc....	6	0	JCK-1
.......................ggaucuuguuugacagcacuc....	14	0	JCK-3
.......................ggaucuuguuugacagcacucu....	4	0	JCK-3
.......................ggaucuuguuugacagcacucu....	5	0	JCK-1
.......................ggaucuuguuugacagcacucu....	6	0	JCK-2
.......................ggaucuuguuugacagcacucA....	2	1	JCK-2
.......................ggaucuuguuugacagcacucucu....	2	0	JCK-2
........................gaucuuguuugacagcacu....	1	0	JCK-2
........................gaucuuguuugacagcacu....	3	0	LT-3
........................gaucuuguuugacagcacu....	14	0	LT-2
........................gaucuuguuugacagcacu....	3	0	JCK-1
........................gaucuuguuugacagcacu....	1	0	LT-1
........................gaucuuguuugacagcacuc....	2	0	LT-1
........................gaucuuguuugacagcacuc....	3	0	LT-3
........................gaucuuguuugacagcacucu....	1	0	LT-1
........................gaucuuguuugacagcacucA....	1	1	LT-1
........................gaucuuguuugacagcacucuc....	4	0	JCK-2
........................gaucuuguuugacagcacucuc....	1	0	JCK-3
........................gaucuuguuugacagcacucuc....	1	0	JCK-1
.........................aucuuguuugacagcacu....	2	0	LT-1
.........................aucuuguuugacagcacu....	3	0	LT-2

```
                         Mature                                                                            Star
uccaucuuguucguuuacccucuggaucuuguuugacagcacucucuuauauuuugccauucaucuuuauaugccaaauggcgaugaugaugauggugggagugcuaucgacaugauccagaaggugcagucgaauaagauacagagggaccacccacaguaccuacucucguuacaaucguauucuucuccucacuuauuauuauuauuauuauuauuccgaucauuucuuuuaaucuauugaag
.........................aucuuguuugacagcacuc	5	0	LT-3
.........................aucuuguuugacagcacuc	2	0	LT-1
.........................aucuuguuugacagcacuc	1	0	LT-2
.........................aucuuguuugacagcacuc	1	0	JCK-2
.........................aucuuguuugacagcacuU	1	1	LT-2
.........................aucuuguuugacagcacuc	8	0	LCK-3
.........................aucuuguuugacagcacucu	3	0	JCK-1
.........................aucuuguuugacagcacucA	7	1	LCK-3
.........................aucuuguuugacagcacucu	4	0	JCK-3
.........................aucuuguuugacagcacucA	2	1	LT-1
.........................aucuuguuugacagcacucu	24	0	LCK-3
.........................aucuuguuugacagcacucu	15	0	JCK-2
.........................aucuuguuugacagcacuUu	1	1	LT-1
.........................aucuuguuugacagcacuUu	1	1	LT-3
.........................aucuuguuugacagcacucu	4	0	LT-1
.........................aucuuguuugacagcacucu	4	0	LT-3
.........................aucuuguuugacagcacucA	1	1	LT-2
.........................aucuuguuugacagcacucu	2	0	LT-2
.........................aucuuguuugCcagcacucu	1	1	JCK-2
.........................aucuuguuugacagcacucuU	6	1	LCK-3
.........................aucuuguuugacagcacucuc	11	0	JCK-3
.........................aucuuguuugacagcacucuc	12	0	JCK-2
.........................Cucuuguuugacagcacucuc	1	1	JCK-2
.........................aucuuguuugacagcacucuA	1	1	JCK-3
.........................aucuuguuugacagcacucuA	3	1	JCK-1
.........................aucuuguuugacagcacucuU	4	1	LT-2
.........................aucuuguuugacagcacucuc	8	0	JCK-1
.........................aucuuguuugacagcacucCcu	1	1	JCK-2
.........................aucuuguuugacagcacucCcu	1	1	LT-3
.........................aucuuguuugacGgcacucucu	1	1	JCK-3
.........................aucuuguuugacagcacucucG	1	1	JCK-1
.........................aucuuguuugacagcacucCcu	1	1	JCK-1
.........................aucuuguuugCcagcacucucu	1	1	JCK-1
.........................Cucuuguuugacagcacucucu	1	1	JCK-1
.........................aucuuguuugacagcacucucu	70	0	JCK-1
.........................aucuuguuugacagcacucucu	1	0	LT-1
.........................aucuuguuugacagcacucucu	107	0	JCK-2
.........................aucuuguuugacagcacucucu	92	0	JCK-3
.........................aucuuguuugacagcacucCcu	1	1	JCK-3
.........................aucuuguuCgacagcacucucuu	1	1	JCK-1
.........................aucuuguuugacagcacucucuA	1	1	JCK-2
.........................aucuuguuugacagcacucucuA	1	1	JCK-1
.........................aucuuguuugacagcacucucuuaAau	1	1	JCK-1
.........................aucuuguuugacagcacucucuuaAau	1	1	JCK-2
..........................ucuuguuugacagcacuc	1	0	LT-2
..........................ucuuguuugacagcacuc	2	0	LT-3
..........................ucuuguuugacagcacucu	2	0	LT-1
..........................ucuuguuugacagcacucA	1	1	LT-3
..........................ucuuguuugacagcacucu	2	0	JT-2
..........................ucuuguuugacagcacucu	2	0	JCK-2
..........................ucuuguuugacagcacucuc	1	0	JCK-3
..........................ucuuguuugacagcacucuc	2	0	JCK-2
..........................ucuuguuugacagcacucucu	1	0	LT-1
..........................ucuuguuugacagcacucucu	6	0	JCK-3
..........................ucuuguuugacagcacucucu	3	0	JCK-1
..........................ucuuguuugacagcacucucu	2	0	LT-3
..........................ucuuguuugacagcacucucu	13	0	JCK-2
..........................ucuuguuugacagcacucucuCa	1	1	LT-1
...........................................................................aaauggUgaugaugaugaug	2	1	JCK-3
.............................................................................auggcgaugaAgaugaug	1	1	LT-1
..............................................................................................gAugggagugcuaucgacaugauc	2	1	LT-1
...............................................................................................Augggagugcuaucgacaugau	1	1	LT-3
.................................................................................................gggagugcuGucgacaugauccagaaggug	1	1	JCK-3
....................................................................................................agugcuaucgacaugauccaga	1	0	LT-3
.....................................................................................................gugcuaucgaAaugaucca	1	1	JCK-2
.....................................................................................................gugcuaucgacaugaucca	1	0	LT-1
.....................................................................................................gugcuaucgacaugauccag	2	0	LT-1
.....................................................................................................gugcuaucgacaugauccag	1	0	LT-2
.....................................................................................................gugcuaucgacaugauccag	1	0	JCK-1
.....................................................................................................gugcuaucgacaugauccagC	1	1	LT-2
```

```
                      Mature                                                                          Star
uccaucuuguucguuuacccucuggaucuuguuugacagcacucucuuauauuuugccauucaucuuuauaugccaaauggcgaugaugaugaugguggga gugcuaucgacaugauccagaaggugcagucgaauaagauacagagggaccacccacaguaccuacucucguuacaaucguauucuucuccucacuuauuauuauuauuauuauuccgaucauuuucuuuuaaucuauugaag
.....................................................................................................gugcuaucgacaugauccaga.............................................................................................................................   12   0   LT-2
.....................................................................................................gugcuaucgacaugauccaga.............................................................................................................................   14   0   LT-3
.....................................................................................................gugcuaucgacaugauccaga.............................................................................................................................   6    0   JCK-1
.....................................................................................................gugcuaucgacaugauccaga.............................................................................................................................   4    0   JCK-2
.....................................................................................................gugcuaucgacaugauccaga.............................................................................................................................   36   0   LCK-3
.....................................................................................................gugcuaucgacaugauccaga.............................................................................................................................   6    0   LT-1
.....................................................................................................Augcuaucgacaugauccaga.............................................................................................................................   1    1   JCK-2
.....................................................................................................gugcuaucgacaugauccaga.............................................................................................................................   3    0   JCK-3
......................................................................................................ugcuaucgacCugauccag..............................................................................................................................   1    1   LT-1
......................................................................................................ugcuaucgacaugauccag..............................................................................................................................   2    0   LT-1
......................................................................................................ugcuaucgacaugauccaga.............................................................................................................................   16   0   JCK-2
......................................................................................................ugcuaucgacaugauccaga.............................................................................................................................   2    0   LT-3
......................................................................................................ugcuaucgacaugauccaga.............................................................................................................................   5    0   JCK-1
......................................................................................................ugcuaucgacaugauccaga.............................................................................................................................   6    0   JCK-3
......................................................................................................uNcuaucgacaugauccaga.............................................................................................................................   1    1   JCK-1
...........................................................................................................ucgacaAgauccagaaggugc.......................................................................................................................   1    1   JCK-2
...........................................................................................................ucgacaAgauccagaaggugc.......................................................................................................................   3    1   JCK-3
...........................................................................................................ucgacaAgauccagaaggugc.......................................................................................................................   2    1   JCK-1
```
